# Tailoring Chlorthalidone Aqueous Solubility by Cocrystallization: Stability and Dissolution Behavior of a Novel Chlorthalidone-Caffeine Cocrystal

**DOI:** 10.3390/pharmaceutics14020334

**Published:** 2022-01-30

**Authors:** Christian Rodríguez-Ruiz, Pedro Montes-Tolentino, Jorge Guillermo Domínguez-Chávez, Hugo Morales-Rojas, Herbert Höpfl, Dea Herrera-Ruiz

**Affiliations:** 1 Facultad de Farmacia, Universidad Autónoma del Estado de Morelos, Av. Universidad 1001, Cuernavaca 62209, Mexico; christian.rodriguezrui@uaem.edu.mx; 2 Centro de Investigaciones Químicas, Instituto de Investigación en Ciencias Básicas y Aplicadas, Universidad Autónoma del Estado de Morelos, Av. Universidad 1001, Cuernavaca 62209, Mexico; pedro.montes@uaem.mx (P.M.-T.); hugom@uaem.mx (H.M.-R.); 3 Facultad de Bioanálisis Región Veracruz, Universidad Veracruzana, Agustín de Iturbide, Veracruz 91700, Mexico; jorgedominguez@uv.mx

**Keywords:** chlorthalidone, pharmaceutical cocrystal, solubility and dissolution studies, cellulosic polymer, X-ray diffraction analysis

## Abstract

A cocrystal of the antihypertensive drug chlorthalidone (CTD) with caffeine (CAF) was obtained (CTD-CAF) by the slurry method, for which a 2:1 stoichiometric ratio was found by powder and single-crystal X-ray diffraction analysis. Cocrystal CTD-CAF showed a supramolecular organization in which CAF molecules are embedded in channels of a 3D network of CTD molecules. The advantage of the cocrystal in comparison to CTD is reflected in a threefold solubility increase and in the dose/solubility ratios, which diminished from near-unit values for *D*_0D_ to 0.29 for *D*_0CC_. Furthermore, dissolution experiments under non-sink conditions showed improved performance of CTD-CAF compared with pure CTD. Subsequent studies showed that CTD-CAF cocrystals transform to CTD form I where CTD precipitation inhibition could be achieved in the presence of pre-dissolved polymer HPMC 80–120 cPs, maintaining supersaturation drug concentrations for at least 180 min. Finally, dissolution experiments under sink conditions unveiled that the CTD-CAF cocrystal induced, in pH-independent manner, faster and more complete CTD dissolution when compared to commercial tablets of CTD. Due to the stability and dissolution behavior of the novel CTD-CAF cocrystal, it could be used to develop solid dosage forms using a lower CTD dose to obtain the same therapeutic response and fewer adverse effects.

## 1. Introduction

**Figure 1:**
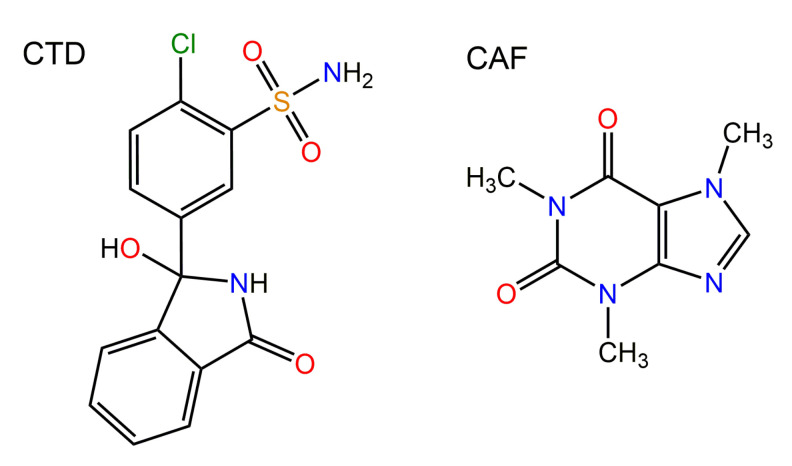
Chemical drawings of the molecular structures of chlorthalidone (CTD) and caffeine (CAF).

Chlorthalidone (CTD) (Figure 1) is a diuretic drug employed for treating hypertension [1,2] and edema associated with congestive heart failure [3]. Because of its long duration of action, CTD is the drug of choice for the treatment of these diseases and prescribed in a dose from 25 to 100 mg/day for controlling hypertension [4,5] whilst for edema a dose from 50 to 200 mg/day is given. CTD has low aqueous solubility (0.191 mg/mL, 0.56 mM in water) [6]. Having a bioavailability of 64% [7], CTD is considered as class IV drug within the biopharmaceutical classification system (BCS) [8,9]. With a pKa of 9.36 [10], CTD is a weak acid and essentially non-ionized in the pH range encompassing the gastrointestinal tract (pH 1–7). In addition to the solubility and absorption problems, CTD has clinically relevant side effects related to decreasing potassium, sodium, and chloride serum levels [11,12,13,14].

Improving the solubility and dissolution rate of an orally administered drug can induce a higher amount dissolved in the gastrointestinal tract, and therefore, a more significant amount of drug is available for absorption into the bloodstream [15,16]. Diverse strategies have been developed to increase the dissolution of CTD, including reduction of the particle size [17], formation of inclusion complexes with β-cyclodextrin [18], microencapsulation by spray-drying and melt granulation coating [19], and incorporation into a solid self-micro-emulsifying drug delivery system (S-SMEDDS) [20]. In addition, the generation of amorphous forms by spray-drying with and without polymers was explored [21,22]. Even though CTD dissolution is improved with these strategies, most involve unit operations raising the production costs or represent challenges for scaling to an industrial level.

An alternative for improving dissolution and other properties of drugs not suitable for salt formation is the search for novel crystalline phases, such as polymorphs, hydrates, solvates, or cocrystals. For CTD, there are reports on four solid crystalline phases, of which polymorph I (REFCODE in the Cambridge Structural Database version 2020.3.0: YUCCIJ) [23] is the most stable phase and constitutes the solid form of CTD in the commercial reference product [6]. CTD form I has an aqueous solubility of only 0.56 mM at 25 °C [6]. Polymorph III (YUCCIJ01) [23] is a conformational polymorph of form I, and hence, might have similar solubility (data not reported yet). Polymorph II (YUCCIJ03) [24] comprises a crystal conglomerate of (*R*)- and (*S*)-enantiomer and is obtained by spontaneous chiral resolution from an equimolar solution in water [24]. Although polymorph II exhibits slightly better solubility (0.83 mM in water at 25 °C [6]) and dissolution rate than polymorph I, scaling the production of polymorph II to large amounts is challenging [6,24]. In addition, there is a report on a chloroform solvate of CTD (GIBMIP) [25], but due to the toxicity of the solvent this phase is not a candidate for pharmaceutical applications. Recently, a drug-drug cocrystal of CTD with the antihypertensive agent betaxolol was proposed on the basis of powder X-ray diffraction (PXRD) analysis, but no further experimental characterization was provided to confirm the composition and to assess physicochemical and biopharmaceutical properties such as solubility, stability, and dissolution rate [26].

Pharmaceutical cocrystals are multi-component molecular crystals in a well-defined stoichiometric ratio composed of an active pharmaceutical ingredient (API) in conjunction with a coformer species. In the crystal structure of cocrystals, the molecular components are interconnected by non-covalent interactions [27,28], in which hydrogen bonds engage frequently as primordial junctions between the components, giving ideally well-defined supramolecular synthons [29,30,31]. Cocrystals have been shown to solve specific drug issues related to physicochemical properties, dosage formulation, and processability, such as stability problems when exposed to elevated temperatures, humidity or light, low solubility or dissolution rates, and deficient compaction properties. In various instances, pharmaceutical cocrystals behave as supersaturable solids displaying significant solubility and dissolution advantages compared to the pristine drug [32,33,34]. However, supersaturated solutions of such cocrystals are prone to convert back to the less soluble drug form and it is still a challenge to control the cocrystal dissolution-supersaturation-precipitation process. In the efforts to modulate this behavior, coformer selection and the use of additives are key aspects [15,35,36].

Caffeine (CAF) is a methylxanthine naturally found in a variety of plants distributed worldwide. CAF is found naturally in many foods, primarily in coffee (27–200 mg/cup) and tea (40–120 mg/cup). CAF is also used as a food ingredient, and in combination with analgesics in over the counter pharmaceutical preparations. Toxicity of methylxanthines in humans is relatively low and moderate consumption is associated with health benefits. Safety thresholds for caffeine have been reported as 400 mg/day for adults, 300 mg/day for pregnant women, <2.5 mg/kg/day for children and adolescents [37]. CAF has been related with a small increase of human blood pressure at moderate intakes ranging from 100 to 400 mg/day for adults [37,38]. This effect is considered mild, transient, and reversible [39]. Currently, no specific recommendation regarding coffee or caffeine intake is present in hypertension guidelines [40]. CAF is one of the most common molecules used to obtain cocrystalline solids [41,42].

In this research work, we report the discovery of a 2:1 CTD cocrystal with CAF (Figure 1) as coformer, for which the solid-state properties were comprehensively analyzed by infrared spectroscopic, calorimetric studies (TGA and DSC) as well as single-crystal X-ray diffraction analysis to elucidate the supramolecular connectivity. The cocrystal undergoes phase transformation upon contact with aqueous solutions, therefore, its solubility (*S*_CC_) and solubility advantage (*SA* = (*S*_CC_/*S*_drug_)) were evaluated by measuring the eutectic constant (*K*_eu_). These thermodynamic indicators and dissolution studies under non-sink and sink conditions demonstrated a superior solubilization of CTD-CAF in comparison to the pristine active pharmaceutical ingredient (API). Considering the critical concentration of CTD for precipitation from the bulk phase (*C*_crit,BP_), the phase transformation to CTD polymorph I observed during the non-sink dissolution studies is proposed to occur at the particle surface. Additionally, the presence of additives enabled to maintain CTD supersaturation levels long enough to be significant for promoting absorption.

## 2. Materials, Preparative Methods, and Characterization Techniques

### 2.1. Materials

Chlorthalidone (99% purity) was obtained from Shaanxi Dideu Medichem Co. Ltd. (Shaanxi, China). Anhydrous Caffeine (98.5–100% purity) was obtained from Productos Químicos Monterrey (Monterrey, Mexico). Copovidone (Kollidon VA^®^ 64 Fine) and polyvinylpyrrolidone (Kollidon^®^ 90) were obtained from BASF Chemical Co. (Ludwigshafen, Germany). Methylcellulose (Methocel^®^ A15), hydroxypropyl methylcellulose (Methocel^®^ E5LV, Methocel^®^ E15LV, and Methocel^®^ E50LV) were kindly donated by Colorcon Inc. (West Point, PA, USA). Hydroxypropyl methylcellulose (HPMC 80–120 cPs), methylcellulose (Methocel^®^ 60 HG), hydroxypropyl cellulose (HPC 80,000, and HPC 370,000), polyvinylpyrrolidone (Kollidon^®^ 25), poloxamer (Kolliphor^®^ P 188 and Kolliphor^®^ P 407), monobasic potassium phosphate (99% purity), sodium hydroxide (97% purity), and HPLC-grade methanol were obtained from Sigma-Aldrich Co. (St. Louis, MO, USA). Hydrochloric acid, absolute ethanol, and dibasic sodium phosphate (99% purity) were obtained from J.T. Baker (Philipsburg, NJ, USA). All other chemicals and the solvents were analytical or reagent grade and used as received without further purification. Ultra-pure water obtained from a Classic UVF water purification system (ELGA LabWater Ltd., High Wycombe, Bucks, UK) was used in this study.

### 2.2. Methods

#### 2.2.1. Preparation of Cocrystal CTD-CAF

For the preparation of cocrystal CTD-CAF, slurry experiments [43] were performed at room temperature using the following general procedure: 50 mg of a 2:1 stoichiometric mixture of CTD and CAF in 60 μL of absolute ethanol were placed in a 5 mL vial and stirred on a Corning^®^ PC-420D stirring hot plate (Chelmsford, Essex, UK) at 550 rpm for 24 h at room temperature. The procedure used for the preparation of larger amounts of cocrystal CTD-CAF consisted in scale-up of the 50 mg preparation technique: 1.554 g (4.6 mmol) of CTD, 0.446 g (2.3 mmol) of CAF, and 2.4 mL of absolute ethanol were placed in a 15 mL vial and stirred for 24 h at 550 rpm. The paste formed was then dried on filter paper for 12 h at room temperature. Cocrystal formation was verified by powder X-ray diffraction analysis.

For the growth of single-crystals of CTD-CAF suitable for SCXRD analysis, crystallization experiments based on slow solvent evaporation were performed: 50 mg of mixtures of CTD and CAF in 2:1, 1:1, 1:2, and 1:3 stoichiometry were dissolved in 2.5 mL of absolute ethanol at 60 °C. After filtration, the solutions were left for slow solvent evaporation at room temperature. After two weeks, single crystals of CTD-CAF were isolated from the 2:1 and 1:1 solutions.

#### 2.2.2. Solid-State Stability Tests

For the solid-state stability experiments, 30 mg of the drug, coformer, cocrystal, and the 1:1 blend of cocrystal with a polymer were introduced into climate-simulating chambers, under the following conditions that simulate environmental storage: (a) 40 °C/0% relative humidity (RH) in a Revco Incubator Chamber RI-23-1060-ABA (Thermo Scientific, Asheville, NC, USA); (b) 50 °C/0% RH in a Rios Rocha Chamber E0-51 (RIOSSA Company, Mexico City, Mexico), and (c) 40 °C/75% RH in a Binder Climatic Chamber IP 20 (BINDER GmbH, Tuttlingen, Germany). The samples were withdrawn after one month and analyzed by PXRD to detect phase changes. All experiments were conducted in duplicate.

#### 2.2.3. Solubility Studies

Drug solubility (*S*_drug_) was measured by adding excess solid to 3 mL of each solubility medium: 60 mM HCl, pH 1.2 and 50 mM phosphate buffer solution (PBS), pH 6.8. The suspensions obtained were magnetically stirred and kept in a water bath at 37.0 ± 0.5 °C for 48 h. A total 1 mL aliquots of the suspension were sampled and filtered through Swinnex^®^ filter holders with Whatman^®^ filter paper grade 3. The 100 µL samples were then diluted to 10 mL with a 50/50 (*v*/*v*) water/methanol mixture. Concentrations of CTD were analyzed by HPLC. All experiments were conducted in triplicate.

Cocrystal solubility (*S*_CC_) and eutectic constant (*K*_eu_) were determined at the eutectic point, where the drug and cocrystal solid phases are in equilibrium with the solution [44]. The eutectic point was approached by a cocrystal dissolution experiment; 60 mg of CTD-CAF and 30 mg of CTD were suspended in 3 mL of each dissolution medium and stirred at 550 rpm for 48 h at 37.0 ± 0.5 °C. Subsequently, 1 mL aliquots were taken and filtered through Swinnex^®^ filter holders with Whatman^®^ filter paper grade 3, and the pH values were measured. The 100 µL samples were then diluted to 10 mL with a 50/50 (*v*/*v*) water/methanol mixture. The recovered solid phases were analyzed by PXRD to determine that both drug and cocrystal solid phases were present, and the filtered solutions were then analyzed by HPLC. All experiments were conducted in triplicate.

According to Kuminek, et al. [45], the solubility (*S*_CC_) of a cocrystal with 2:1 stoichiometry ratio can be calculated with Equation (1):

(1) $$S_{\mathrm{CC}}^{2:1}=2\left(\sqrt[{3}]{\frac{{\left[\mathrm{drug}\right]}_{T,\mathrm{eu}}^{2}{\left[\mathrm{coformer}\right]}_{T,\mathrm{eu}}}{4}}\right)$$

where [drug]*_T_*_,eu_ and [coformer]*_T_*_,eu_ are the experimental molar concentrations of drug and coformer at the eutectic point, respectively. With the cocrystal solubility value, the eutectic constant for a 2:1 cocrystal can be obtained as follows (Equation (2)) [45]:

(2) $$K_{\mathrm{eu}}^{2:1}=0.5{\left(\frac{S_{\mathrm{CC}}}{S_{\mathrm{drug}}}\right)}^{3}$$

where *S*_CC_ and *S*_drug_ are the cocrystal and drug molar solubilities. The solubility advantage (*SA*) is calculated by Equation (3) [44]:

(3) $$SA=\frac{S_{\mathrm{CC}}}{S_{\mathrm{drug}}}$$

Once the solubility values of the drug (*S*_drug_) and the cocrystal (*S*_CC_) are known, the drug dose/solubility (*D*_0D_) and cocrystal dose/solubility (*D*_0CC_) ratios are calculated using Equations (4) and (5) [36]. Where the molar dose concentration (*C*_dose_) is the value defined by the drug marketed dose of CTD (50 mg = 0.148 mmol) divided by the luminal volume (250 mL).

(4) $$D_{0D}={\frac{C_{\mathrm{dose}}}{S_{\mathrm{drug}}}}_{}$$

(5) $$D_{0\mathrm{CC}}={\frac{C_{\mathrm{dose}}}{S_{\mathrm{CC}}}}_{}$$

#### 2.2.4. Polymer Selection

The solvent-shift experiments were performed according to the procedure reported in the literature [35,46]. Thirteen candidate polymers were evaluated to prove their ability to maintain supersaturated CTD concentrations: HPC 80,000 cPs, HPC 370,000 cPs, HPMC 80–120 cPs, Kollidon^®^ VA64 Fine, Kollidon^®^ 25, Kollidon^®^ 90, Kolliphor^®^ P 188, Kolliphor^®^ P 407, Methocel^®^ 60 HG, Methocel^®^ A15, Methocel^®^ E5LV, Methocel^®^ E15LV, and Methocel^®^ E50LV (see Table S2, for further polymer details). A stock solution of CTD was generated by dissolving 200 mg in 1 mL of DMSO (590.38 mM). Then, 0.5% *w*/*v* of each polymer was dissolved in HCl pH 1.2 or PBS pH 6.8; as a reference, each dissolution medium without polymer was evaluated. A volume of 2.5 mL of each medium, with or without polymer, was placed in a spectrophotometer quartz cell, followed by stepwise addition of aliquots of 10 µL each from CTD stock solution in DMSO. After stirring for 5 min, the absorbance of the solution was measured in the UV-Vis spectrophotometer (Varian UV Cary 50 spectrophotometer, Palo Alto, CA, USA) at λ = 550 nm (where CTD does not absorb) for each data point. The UV-Vis spectrum baseline increases with suspended particles (precipitation) appearing in the medium due to light scattering. The polymer performance is effective if CTD concentration is maintained as high as possible before observing precipitation. All experiments were conducted in triplicate.

#### 2.2.5. Powder Dissolution under Non-Sink Conditions and Phase Stability

For powder dissolution experiments, 155 mg of CTD, 200 mg of cocrystal (equivalent to 155 mg of CTD), or 200 mg of a physical mixture of CTD and CAF in 2:1 molar ratio were used. The powders were grounded in an agate mortar using a pestle, passed through a sieve mesh 200 to eliminate agglomerated particles, added to 10 mL of HCl pH 1.2 or PBS pH 6.8 at 37 ± 0.5 °C and stirred at 90 rpm in a Personal Reaction Station (J-Kem Scientific Inc., St. Louis, MO, USA). Aliquots of 1.0 mL with medium reposition were taken with a syringe every minute during 10 min, every two minutes from 10 to 30 min, and at 45, 60, 90, 120, and 180 min. The aliquots were filtered through Swinnex^®^ filter holders with Whatman^®^ filter paper grade 3. 100–200 µL samples were then diluted to 5–10 mL with a 50/50 (*v*/*v*) methanol/water mixture. The solution concentrations of the drug and coformer were analyzed by HPLC, whilst the powder retained in the filter was analyzed by PXRD for examining phase stability. Similarly, dissolution experiments with HCl pH 1.2 and PBS pH 6.8 solutions containing HPMC 80–120 cPs pre-dissolved at 0.5% *w*/*v* (polymer selected by solvent shift method, see Section 3.7) were performed. All experiments were conducted in triplicate.

#### 2.2.6. Induced Precipitation Experiments

Induced precipitation experiments were performed according to the procedure described by Omori, et al. [47], using a Personal Reaction Station (J-Kem Scientific Inc., St. Louis, MO, USA). A stock solution of CTD was generated by dissolving 200 mg in 1 mL of DMSO (590.4 mM). Aliquots were added to 20 mL of HCl pH 1.2 or PBS pH 6.8 at 37 °C stirred at 90 rpm, to obtain CTD solutions with the concentrations 3.2, 3.5, 3.8, 4.1, and 4.4 mM. From each solution samples of 1 mL were withdrawn at minutes 3, 6, 10, 15, 20, 30, 45, 60, 90, 120, and 180 without medium reposition and filtered through Swinnex^®^ filter holders with Whatman^®^ filter paper grade 3. Aliquots of 100 μL were then diluted with a 50/50 (*v*/*v*) water/methanol mixture and analyzed by HPLC to measure the solution concentrations of CTD. All the experiments were conducted in triplicate.

#### 2.2.7. Preparation of Capsule Formulations

For dissolution experiments under sink conditions, capsule formulations were prepared containing physical mixtures of CTD-CAF and HPMC 80–120 cPs. Formulations of CTD-CAF with HPMC 80–120 cPs were obtained considering 50 mg of CTD dose in the powder blend. The amount of polymer used corresponds to 0.0, 2.0, 5.0, or 10.0% *w*/*w* considering the average tablet weight (*n* = 20, 141.48 ± 0.62 mg) of the commercially available CTD reference product in Mexico, Higroton^®^ 50 (50 mg dose) (Table 1). Cocrystal and polymer powders were mixed for 3 min in glass vials; the blends were ground with mortar and pestle and passed through a sieve mesh 200 to eliminate agglomerates. Then, the powders were remixed for 3 min and formulations 1–4 filled into hard gelatin capsules #4.

For a reference experiment, capsules with powder of commercially available CTD tablets were also prepared. The tablets were ground with a mortar and pestle and passed through a sieve mesh 200, whereupon hard gelatin capsules #4 were filled with 141.5 mg of powder equivalent to 50 mg of CTD.

#### 2.2.8. Dissolution Experiments under Sink Conditions

The dissolution experiments under sink conditions were performed using an Agilent 708-DS Dissolution Apparatus USP 1 (Santa Clara, CA, USA). Baskets with the capsules were placed in vessels with 500 mL of HCl pH 1.2 or PBS pH 6.8 at 37 ± 0.5 °C, and stirred at 100 rpm. Aliquots of 3 mL were taken at 5, 10, 15, 20, 30, 45, 60, 90, and 120 min, and filtered through Swinnex^®^ filter holders with Whatman^®^ filter paper grade 3. The volume was replaced immediately after each sample. The 50–100 µL aliquots were then diluted to 5 to 10 mL with a 50/50 (*v*/*v*) methanol/water mixture and analyzed by HPLC with UV detection to quantify the CTD and CAF concentrations. The solid residues collected at the end of the dissolution tests were dried at room temperature and analyzed by PXRD. All experiments were conducted in triplicate. The percentage of CTD dissolved was calculated as:

$$\mathrm{mg}\;\mathrm{of}\;\mathrm{CTD}\mathrm{dissolved}\;=\;C_{n}\times C_{n}+\;\sum_{i=1}^{n-1}C_{i}\times V_{s}\;\%\;\mathrm{CTD}\;\mathrm{dissolved}\;=\;\mathrm{mg}\;\mathrm{of}\;\mathrm{CTD}\;\mathrm{dissolved}\;\times \;100/50\;\mathrm{mg}\;\mathrm{CTD}$$

where *C_n_* = concentration of drug in sample *n*, *V_n_* = volume of dissolution medium at the time of sample *n* withdrawal; *C_i_* = concentration of drug in sample *n* − 1 and *V_s_* = volume of aliquot due to sampling.

### 2.3. Characterization Techniques

#### 2.3.1. Powder X-ray Diffraction Analysis (PXRD)

The solids obtained from the screening experiments for cocrystal phases of CTD were analyzed by powder X-ray diffraction (PXRD) using a BRUKER D8-ADVANCE diffractometer (λ_CuKα1_ = 1.54056 Å, germanium as monochromator) equipped with a LynxEye detector (Karlsruhe, Germany). For the PXRD analysis of the solids recovered from the solubility and dissolution experiments, a BRUKER D2 PHASER 2nd Generation diffractometer (λ_CuKα1_ = 1.54184 Å) equipped with a LynxEye detector (Karlsruhe, Germany) and operated at 30 kV and 10 mA was used. On both diffractometers, data were collected at room temperature in the 2-theta range of 5–45° with a step size of 0.02°.

#### 2.3.2. Single-Crystal X-ray Diffraction Analysis (SCXRD)

For the characterization by single-crystal X-ray diffraction (SCXRD) analysis of CTD-CAF, a Bruker D8 Quest diffractometer (Karlsruhe, Germany) equipped with a CCD area detector (CMOS photon 100) using Cu Kα radiation (*λ* = 1.54178 Å) was employed. Frames of the diffraction patterns were collected at room temperature. The measured intensities were reduced to *F*^2^ and corrected for absorption using spherical harmonics (SADABS) [48]. Structure solution, refinement, and data output were performed with the OLEX2 program package [49] using SHELXT [50] for the structure solution and SHELXL [51] for the refinement. Non-hydrogen atoms were refined anisotropically. C-H atoms were placed in geometrically calculated positions and refined using a riding model, in which each H atom was assigned a fixed isotropic displacement parameter. O-H and N-H hydrogen atoms were localized in difference Fourier maps and then refined with *U*_ij_ and geometry restraints. Figures were created with Diamond [52].

Crystallographic data for the crystal structure have been deposited with the Cambridge Crystallographic Data Centre as supplementary publication no. 2130557. Copies of the data can be obtained free of charge on application to CCDC, 12 Union Road, Cambridge CB2 1EZ, UK (fax: (+44)1223-336-033; e-mail: deposit@ccdc.cam.ac.uk, http://www.ccdc.cam.ac.uk).

#### 2.3.3. Infrared Spectroscopy Analysis (IR)

The IR spectroscopic characterization was performed on a ThermoScientific FT-IR NICOLET 6700 spectrophotometer (Waltham, MA, USA) in the 4000–400 cm^−1^ range, using the Smart iTR accessory with a diamond ATR crystal.

#### 2.3.4. Thermogravimetric Analysis and Differential Scanning Calorimetry (TGA-DSC)

A combined TGA-DSC analysis was realized with a SDT-Q600 simultaneous analyzer apparatus from TA Instruments (New Castle, DE, USA). For the experiment, roughly 3 mg of sample was placed in a 40 µL alumina crucible (non-sealed) and analyzed in the temperature range of 25–600 °C using a heating rate of 10 °C/min and a nitrogen flow of 100 mL/min.

#### 2.3.5. High-Performance Liquid Chromatography (HPLC)

CTD and CAF concentrations were quantified by an Agilent 1260 Infinity HPLC system (Agilent Technologies, Santa Clara, CA, USA) equipped with a DAD detector. An Agilent InfinityLab Poroshell 120 EC-C18 column with a particle size of 2.7 μm, and dimensions of 4.6 × 100 mm was used for separation at 35 °C. The mobile phase was composed of 50% methanol and 50% water, and the flow rate was set at 0.6 mL/min. The injection volume was 15 μL, and the wavelengths used for detection were 226 nm and 273 nm for CTD and CAF, respectively. An analytical method was developed and validated to quantify the CTD and CAF concentrations of the samples of all solubility and dissolution experiments. The parameters validated were accuracy, repeatability, linearity, and range [53]. Standard calibration curves were established in the range of 0.5–16 µg/mL for CTD and 1–25 µg/mL for CAF.

### 2.4. Statistical Analysis

To compare dissolution profiles under non-sink conditions was carried out a two-way analysis of variance (ANOVA) of the area under the curve (AUC, mM min^−1^). A Tukey multiple comparison test was performed with a significance level of 0.05 using the OriginPro 2018 software package (OriginLab Co., Northampton, MA, USA).

## 3. Results

### 3.1. Preparation of Cocrystal CTD-CAF

**Figure 2:**
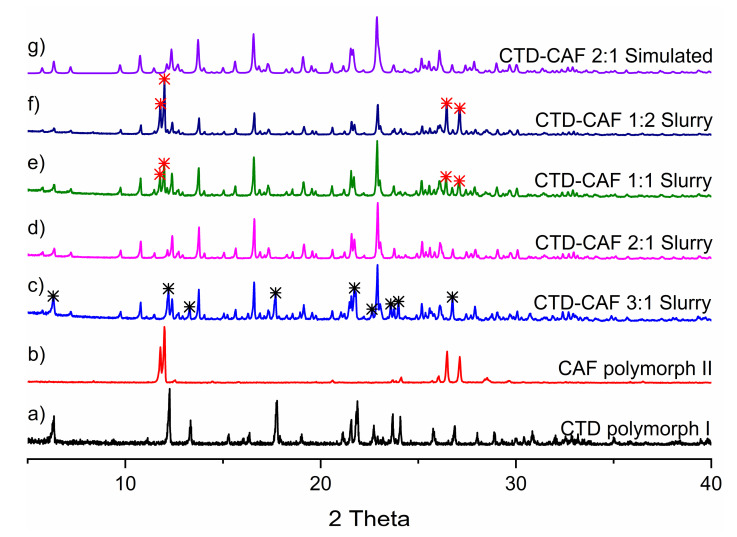
PXRD patterns of (**a**) CTD, (**b**) CAF and CTD-CAF prepared by slurry with ethanol in (**c**) 3:1, (**d**) 2:1, (**e**) 1:1, (**f**) 1:2 stoichiometry. (**g**) PXRD pattern of CTD-CAF simulated from the single-crystal X-ray diffraction analysis. Note: asterisks indicate representative signals of the raw materials in excess; black for CTD and red for CAF.

The chlorthalidone solid used in the present study matches with polymorph I (REFCODE in the Cambridge Structural Database, version 2020.3.0 [54]: YUCCIJ [23]), while the caffeine corresponds to the anhydrous β-caffeine form (polymorph II, NIWFEE03 [55]) (Figure S1). Experiments for obtaining the cocrystal (CTD-CAF) were conducted using the slurry technique, an efficient and widely used method for generating cocrystals with low-soluble drugs [43]. For determining the stoichiometric ratio of CTD and CAF in the cocrystal, the starting reagents were combined in 3:1, 2:1, 1:1, and 1:2 molar ratios, using ethanol, a class 3 solvent with low risk to human health [56]. The slurry products were characterized by PXRD analysis and compared to the previously reported PXRD patterns of the starting materials, as shown in Figure 2. The slurry experiments conducted in 3:1, 1:1, and 1:2 molar ratios of CTD and CAF gave phase mixtures of the cocrystal with CTD and CAF. Only in the product of the 2:1 reaction peaks for the starting reagents were absent (Figure 2d). In addition, the pattern simulated from the SCXRD analysis of the cocrystal (*vide infra*) is in excellent agreement with the PXRD pattern obtained from this experiment.

### 3.2. Crystallographic Analysis

The crystal structure of CTD-CAF was determined by SCXRD analysis on a crystal grown by slow solvent evaporation from a solution in absolute ethanol. The most relevant crystallographic data are summarized in Table 2. Distances and angles for the intermolecular hydrogen bonding interactions in CTD-CAF are given in Table S1.

**Figure 3:**
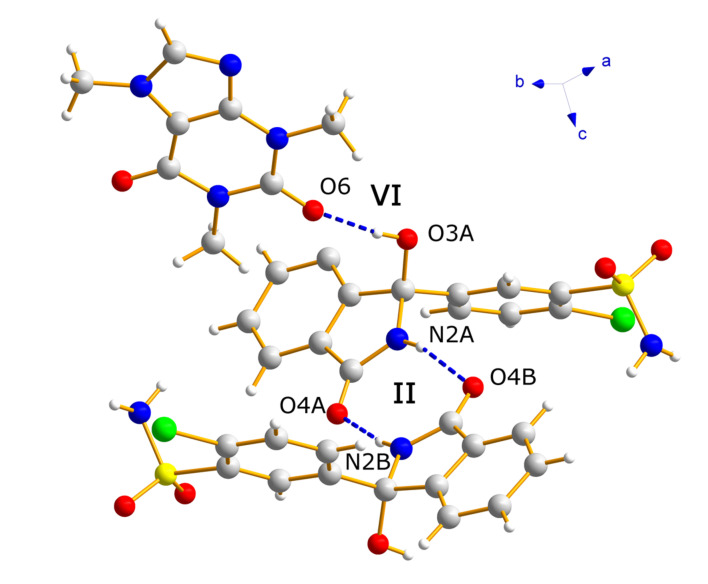
The asymmetric unit of cocrystal CTD-CAF, as determined by single-crystal X-ray diffraction analysis.

**Figure 4:**
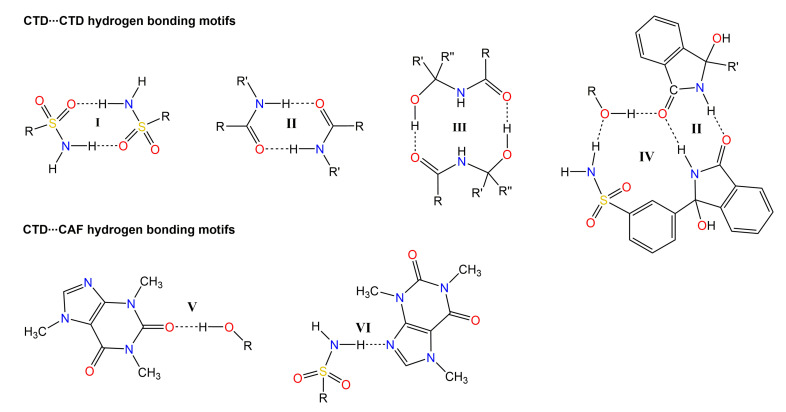
Representation of the relevant hydrogen bonding motifs in the supramolecular arrangement of the crystal structure of CTD-CAF.

**Figure 5:**
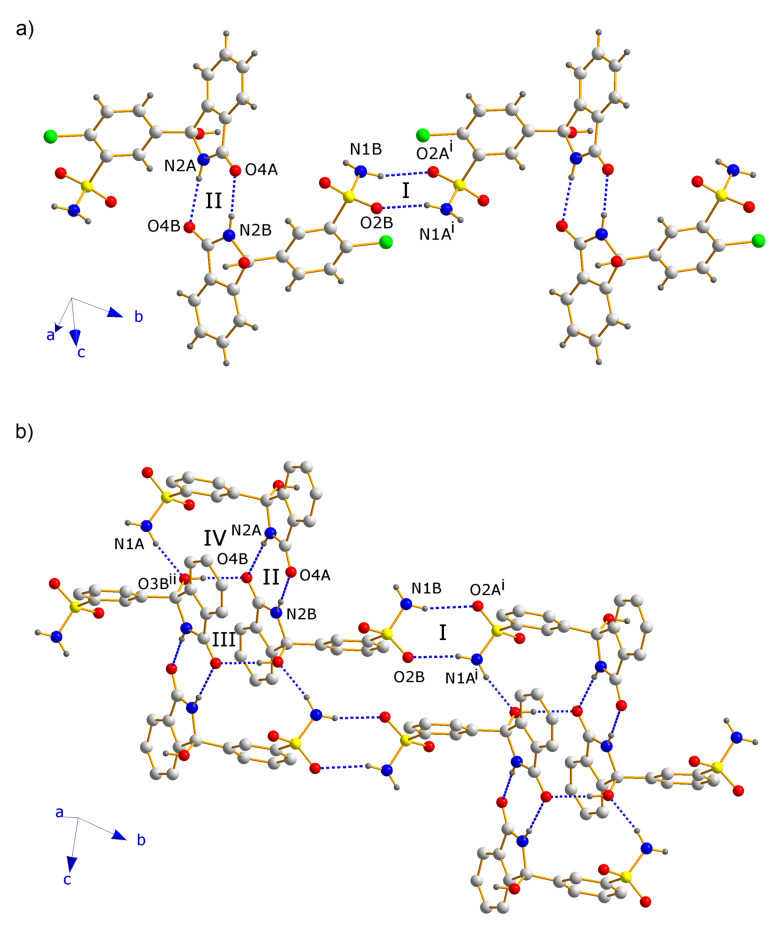
Fragments of the crystal structure of CTD-CAF, showing (**a**) the zig-zag strand formed by CTD molecules interacting through motifs **I** and **II**, and (**b**) the double strands resulting by interconnection through motifs **III** and **IV**. **Note:** For clarity, in Figure 5b C-H hydrogen and chlorine atoms were omitted. Symmetry operators: (**i**) −1 + x, 1 + y, z; (**ii**) 1 − x, 1 − y, 2 − z.

CTD-CAF crystallized in the space group *P*-1, with the asymmetric unit consisting of two crystallographically independent CTD molecules and one CAF molecule (Figure 3). Analysis of the crystal structure reveals a total of six N-H⋯O, O-H⋯O, and N-H⋯N hydrogen-bonding motifs that govern the supramolecular organization of CTD-CAF (Figure 4). In first instance, two homosynthons formed by N-H⋯O type hydrogen bonds (motifs **I** and **II**, Figure 4) link the two crystallographically independent CTD molecules into zig-zag strands running along [−1 1 0] (Figure 5a). Synthons **I** and **II** consist of double-bridged 8-membered rings, which are common in compounds with sulfonamide groups and lactam rings [57,58,59,60]. Adjacent 1D strands are interconnected by additional O-H⋯O and N-H⋯O contacts embedded in two 12-membered H-bonded rings (motifs **III** and **IV**, Figure 4) to yield overall hydrogen bonded double strands. Motif **III** joins pairs of one of the two crystallographically independent CTD molecules where the hydroxyl and carbonyl groups attached to the lactam rings function as donor and acceptor, respectively. Motif **IV** involves three CTD molecules linked through single N-H⋯O and O-H⋯O hydrogen bonds originated from motifs **II** and **III**, and an additional N-H⋯O interaction formed between an N-H_sulfonamide_ group and the oxygen atom of the hydroxyl group involved in motif **III** (Figure 5b).

**Figure 6:**
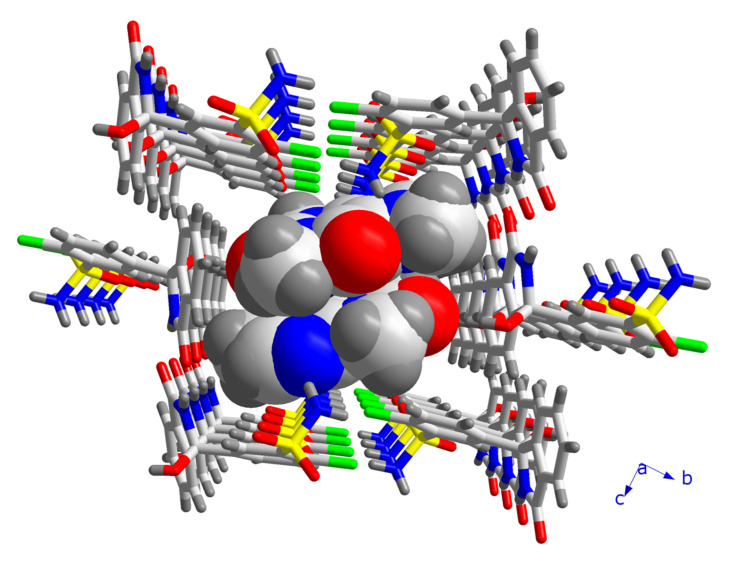
Fragment of the crystal structure of CTD-CAF, illustrating the channels formed by CTD molecules along the *a*-axis, in which pairs of CAF molecules are embedded.

The 1D double strands are packed into a 3D network with channels along the *a*-axis, in which pairs of CAF molecules linked through C-H⋯O contacts are embedded (Figure 6). The CAF molecules are bound to the CTD network by means of single hydrogen bonds of the O-H⋯O (motif **V**) and N-H⋯N type (motif **VI**, Figure 4). This structure resembles a lattice inclusion compound (clathrate) [61], and a somewhat related structural organization was observed in the CTD-solvate with chloroform [25]. Comparing the intermolecular connectivity in the crystal structure of CTD-CAF with the previously reported crystalline phases of CTD (Table 3), the close relationship to polymorph I is noteworthy. CTD-CAF and CTD polymorph I both exhibit the 1D double strands shown in Figure 5b with motifs **I**–**IV** (YUCCIJ) [23], whilst polymorphs II (YUCCIJ03) [24] and III (YUCCIJ01) [23] and the chloroform solvate (GIBMIP) [25] share only one or two motifs with the cocrystal.

### 3.3. Analysis by IR Spectroscopy

**Figure 7:**
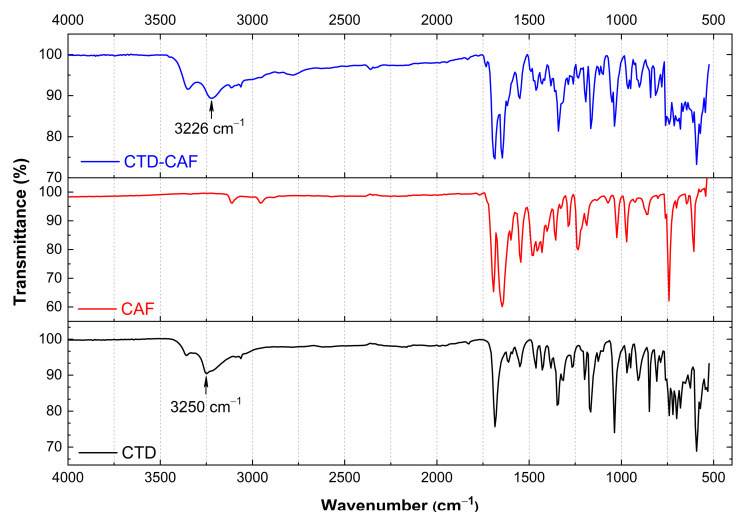
IR spectra of chlorthalidone (CTD polymorph I), caffeine (CAF), and cocrystal CTD-CAF.

Comparison of the solid-state IR spectra of the starting materials and products of cocrystallization experiments allows establishing the formation of a new solid phase since changes in the vibrations of functional groups occur due to changes in the interaction patterns, particularly when hydrogen bonding motifs are varying [62,63,64]. The IR spectra of CTD (polymorph I), CAF, and CTD-CAF are shown in Figure 7, with the IR spectra of CTD and CAF matching with previously reported data [10,65]. The IR spectrum of CTD-CAF reveals characteristic bands of both components with relatively small or negligible differences in the intermolecular CTD⋯CTD binding compared to the starting reagents, which is in agreement with the close relationship of CTD and CTD-CAF (see Section 3.2.). The largest displacement among the IR bands of CTD correspond to the -N-H stretching mode of the CTD lactam group (3250 cm^−1^ in CTD versus 3226 cm^−1^ in the cocrystal). CTD polymorph I comprises a double-bridged hydrogen-bonded synthon between the lactam groups of two drug molecules (motif **II**, Figure 4) which also appears in the crystalline network of CTD-CAF; however, the N-H⋯O distances of 2.819(4) Å and 2.959(4) Å in CTD-CAF are shorter than those present in CTD polymorph I [twice 2.977(3) Å] [23]. The change in the interaction strength originates the shift of the IR band to a smaller wavenumber due to elongation of the N-H bond. The CTD bands at 3351, 1685, 1348, and 592 cm^−1^, corresponding to the -OH, -CONH, -SO_2_NH_2_, and -C-Cl functional groups, do not change their displacement.

### 3.4. TG-DSC Analysis

**Figure 8:**
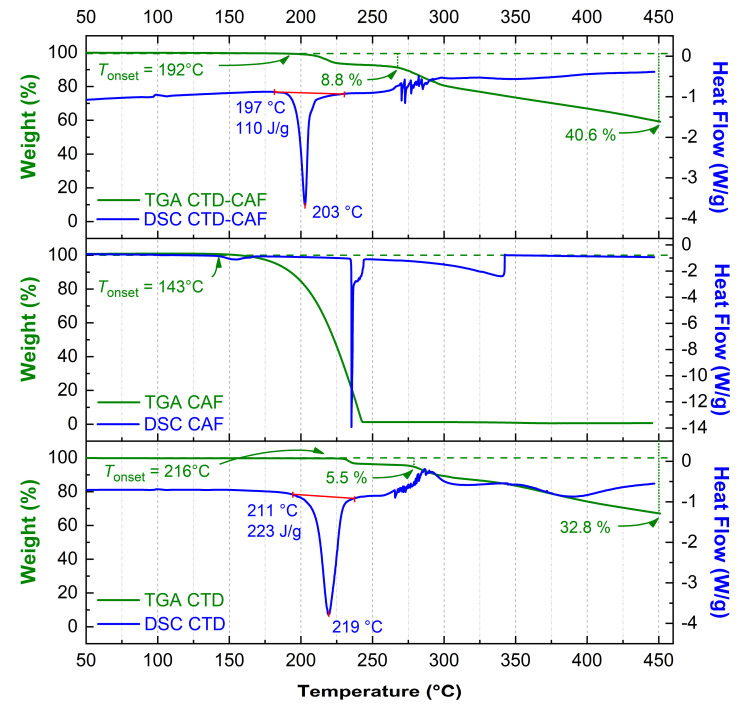
DSC and TGA thermograms for chlorthalidone (CTD polymorph I), caffeine (CAF), and cocrystal CTD-CAF.

DSC and TGA traces of CTD (polymorph I), CAF, and CTD-CAF are shown in Figure 8. The endothermic peak related to the melting point in the trace for CTD (*T*_onset_ = 211 °C; *T*_peak_ = 219 °C; Δ_fus_H^o^ = 75.5 kJ mol^−1^) agrees well with data previously reported in the literature [10,19,65]. After melting, stepwise decomposition of CTD is observed starting at *T*_onset_ = 216 °C with a weight loss of 5.5% (calc. 5.3%) for the first and only well-defined step of mass loss. The decomposition is attributed to dehydroxylation of the tertiary alcohol group (=elimination of water) according to a product identified previously by Bauer et al. in degradation experiments of CTD in acid conditions [66]. For other potential decomposition events such as the elimination of chlorine atoms (calc.: 10.5%) and the SO_3_-derivative SO_2_(NH) (calc.: 23.3%), larger percentage weight losses would be expected. The accumulated percentage weight loss at the end of the experiment at *T* = 450 °C (32.8%) is slightly smaller than the value calculated for the sum of the above-mentioned processes (39.1%), but is in agreement with the still decreasing slope of the curve.

For CAF, a small endothermic peak is observed at 143 °C in the DSC graph, whilst TGA indicates a single-step weight loss starting at *T*_onset_ = 143 °C and ending at 245 °C, which is originated from sublimation [65].

For the cocrystal CTD-CAF, melting (*T*_onset_ = 197 °C; *T*_peak_ = 203 °C; Δ_fus_H^o^ = 95.8 kJ mol^−1^) occurs at lower temperature than for CTD. The TGA reveals a trace similar to CTD with the first and clearly defined step of weight loss initiating at somewhat lower temperature (*T*_onset_ = 192 °C). The 8.8% weight loss exceeds the value expected for dehydroxylation of the CTD molecule equivalents (calc.: 4.1%) and probably involves simultaneous decomposition of CTD and partial elimination of CAF. The accumulated percentage weight loss achieved at the end of the experiment is 40.6%. The difference with respect to pure CTD does not correspond to the elimination of all CAF, indicating that probably a chemical reaction among the residues of CTD and CAF occurred. Thus, for CTD-CAF the cocrystal decomposition process is complex and does not initiate just with coformer release, as observed for other pharmaceutical cocrystals [67,68].

### 3.5. Solid-State Stability Tests

**Figure 9:**
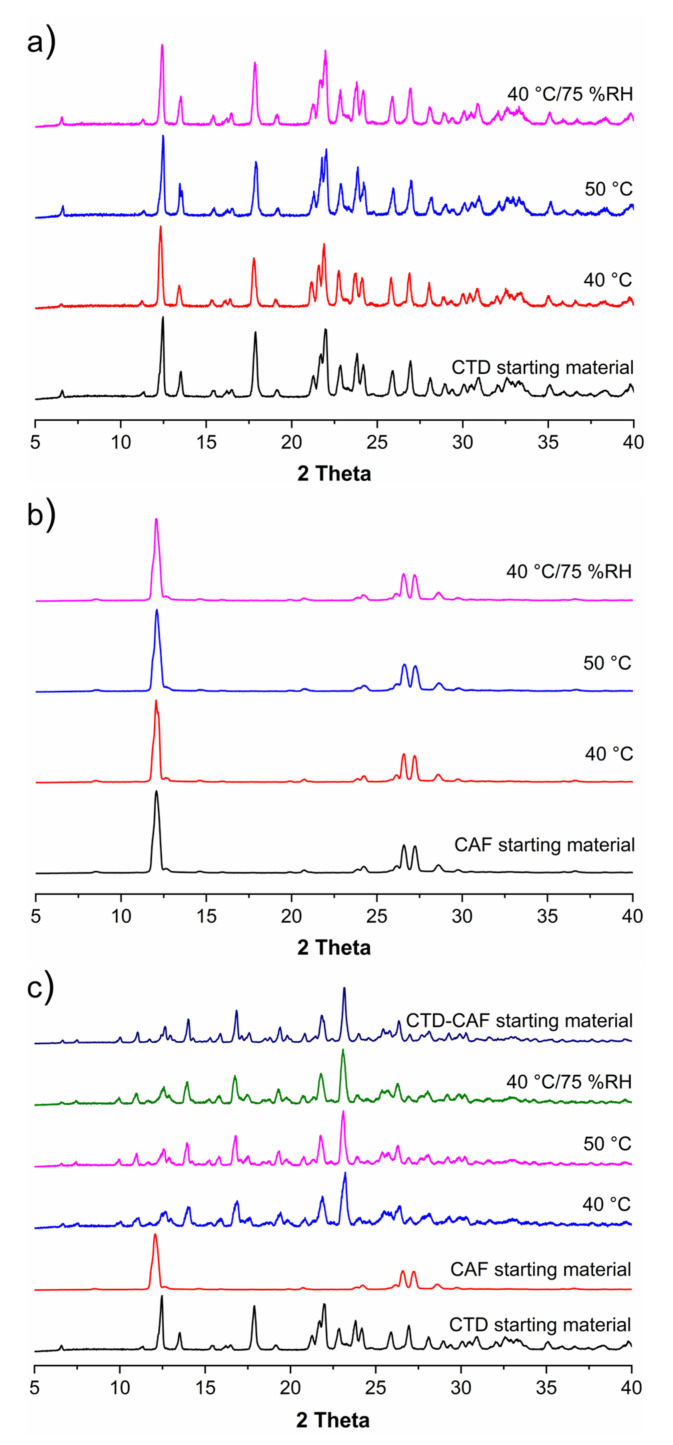
Powder X-ray diffraction (PXRD) patterns of (**a**) CTD, (**b**) CAF and (**c**) CTD-CAF after treatment with temperature and relative humidity (RH) stress conditions at 40 °C, 50 °C, and 40 °C/75% RH.

PXRD patterns obtained for solids of CTD, CAF, and CTD-CAF after a month incubated under temperature and relative humidity stress conditions are shown in Figure 9a–c, respectively. No changes in the PXRD patterns were observed compared to the starting materials, showing that CTD, CAF, and CTD-CAF are physically and chemically stable under the conditions tested.

### 3.6. Solubility Studies

Table 4 summarizes the drug solubility (*S*_drug_), cocrystal solubility (*S*_CC_), eutectic constant (*K*_eu_), solubility advantage (*SA*) and, drug (*D*_0D_) and cocrystal (*D*_0CC_) dose/solubility ratios. The cocrystal solubility was obtained from Equation (1), using the drug and coformer concentrations determined at the eutectic point, where drug and cocrystal solid phases are in equilibrium with the solution [44]. No change of the pH was observed at the end of the experiments. The solubility values of pure CTD at pH 1.2 (0.687 ± 0.005 mM) and pH 6.8 (0.643 ± 0.007 mM) are in agreement with data previously reported by França, et al. [22] at pH 6.8 (0.7 ± 0.01 mM/mL). CTD is a weak acid (p*K*_a_ = 9.36), and therefore, hardly ionized at pH 1.2–6.8 to influence its intrinsic solubility. *S*_CC_ is higher than *S*_drug_ in both dissolution media resulting in *SA* values of 2.91 (pH 1.2) and 3.18 (pH 6.8). The advantage of the cocrystal concerning its solubility in comparison to CTD is reflected also in the dose/solubility ratios, which diminished from near-unit values for *D*_0D_ to 0.29 for *D*_0CC_.

### 3.7. Polymer Selection for Dissolution Studies

**Figure 10:**
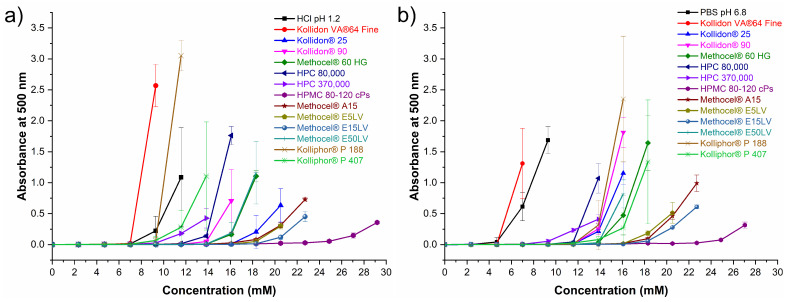
Inhibitory effect of pre-dissolved polymers (0.5% *w*/*v*) on CTD precipitation in (**a**) HCl pH 1.2 and (**b**) BF pH 6.8. (mean ± SD, *n* = 3).

Solvent-shift experiments were carried out to select a polymer with good performance to inhibit or delay nucleation and crystal growth in order to maintain supersaturated CTD concentrations before observing precipitation (i.e., the parachute effect) [69]. The results of the solvent shift experiments are shown in Figure 10. Addition of aliquots from a CTD stock solution (590.3 mM) to 2.5 mL of polymer-free dissolution medium (HCl pH 1.2) caused CTD precipitation at concentrations higher than 7.00 mM; in contrast, at pH 6.8, precipitation initiated at concentrations above 4.69 mM. In acidic dissolution medium with polymer pre-dissolved at 0.5% (*w*/*v*), only Kollidon VA^®^ 64 Fine induced precipitation at a lower concentration than the polymer-free medium; the remaining polymers maintained CTD in solution from 7.00 to 22.7 mM. Of these, the polymer with the best performance in HCl pH 1.2 was HPMC 80–120 cPs. At pH 6.8, all polymers were able to inhibit CTD precipitation at concentrations above 7.00 mM, particularly, HPMC 80–120 cPs kept CTD in solution up to 22.7 mM. Based on these results, HPMC 80–120 cPs was selected for subsequent dissolution experiments under non-sink and sink conditions.

### 3.8. Powder Dissolution Studies under Non-Sink Conditions

**Figure 11:**
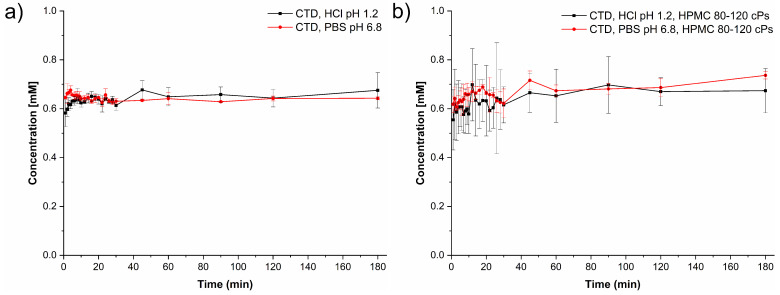
Dissolution profiles of CTD (polymorph I) under non-sink conditions (**a**) in the absence and (**b**) in the presence of HPMC 80–120 cPs pre-dissolved at 0.5% (*w*/*v*), in HCl pH 1.2 (black lines) and PBS pH 6.8 (red lines) (mean ± SD, *n* = 3).

Drug and cocrystal dissolution experiments were performed at pH 1.2 and 6.8 simulating physiological pH gastrointestinal tract conditions in the absence and presence of HPMC 80–120 cPs at 0.5% (*w*/*v*). The pH was constant during the experiments. The dissolution profiles of CTD without polymer given in Figure 11a show that the CTD concentration remained constant throughout the experiments affording an average value of 0.65 mM in both media. A similar result was observed in the presence of pre-dissolved polymer in solution (Figure 11b), indicating that the polymer does not have a solubilizing effect over the drug. The solid samples recovered after the dissolution tests were analyzed by PXRD. The diffraction patterns were identical to CTD, indicating that phase transformation had not occurred under these conditions (Figure S2).

**Figure 12:**
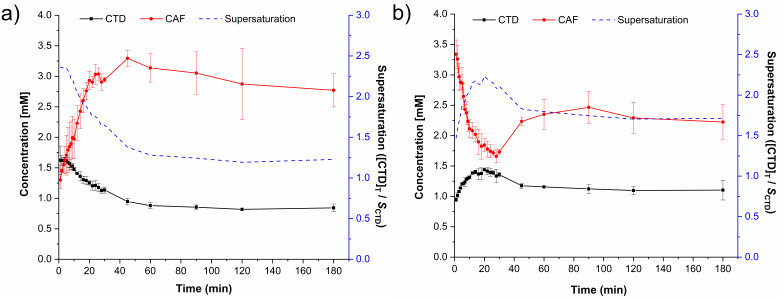
Dissolution profiles of CTD-CAF under non-sink conditions in (**a**) HCl pH 1.2 and (**b**) PBS pH 6.8. Continuous lines represent the dissolution profiles of CTD (black) and CAF (red). Blue dashed lines represent the supersaturation of CTD calculated by dividing the total amount of drug ([CTD]_T_) at each time over *S*_CTD_. Mean ± SD, *n* = 3.

Figure 12 shows the dissolution profiles measured for each of the components from dissolving cocrystal at pH 1.2 and 6.8. The supersaturation of CTD was calculated by dividing each point of the dissolution profile over CTD solubility (Figure 12a, blue dashed line). In the first case (pH 1.2, Figure 12a), CTD supersaturation was observed since the beginning of data acquisition ([CTD]_T_/*S*_CTD_ = 2.36, *t* = 1 min). This condition was maintained for several minutes, decaying over time to a concentration of 0.84 mM, which is 1.2 times the solubility of CTD. In contrast, in the course of the dissolution of CTD-CAF, the CAF concentration increased from 1.30 mM at minute 1 up to a maximum of 3.30 mM at 45 min, whereupon the concentration decayed slowly to 2.77 mM towards the end of the experiment. These CAF concentrations were saturated with respect to the cocrystal, which is the main solid in contact with the solution [69].

The cocrystal component dissolution profiles evaluated at pH 6.8 are presented in Figure 12b. The initial CTD concentration (*t* = 1 min) of 0.95 mM increased up to 1.44 mM within 20 min followed by a decrease to 1.17 mM after 45 min and remaining constant until the end of the experiment. Accordingly, the supersaturation of CTD passes through a maximum of ([CTD]_T_/*S*_CTD_ = 2.23 at *t* = 20 min. Conversely, the initial CAF concentration of 3.34 mM decayed constantly to 1.66 mM (*t* = 28 min) and then grew up to 2.22 mM towards the end of the experiment. Previously, Lim and Go [70] observed a solubilizing concentration-dependent effect of caffeine over the drug halofantrine. To explore if a similar effect occurred here, dissolution experiments were also performed for the physical mixture of CTD and CAF in 2:1 stoichiometry (MF CTD:CAF). The CTD and CAF concentration profiles at pH 1.2 and 6.8 given in Figure S3a,b indicate CTD solutions that are saturated with a constant value during the entire experiment (0.65 mM at pH 1.2 and pH 6.8); CAF dissolved immediately without saturation giving the maximum concentration starting from minute one (17.12 mM) which is more than ten times below its solubility (*S*_CAF_ = 175 mM at pH 1.2, 37 °C). These results indicate that CAF does not have a solubilizing effect over CTD under this experimental setting.

**Figure 13:**
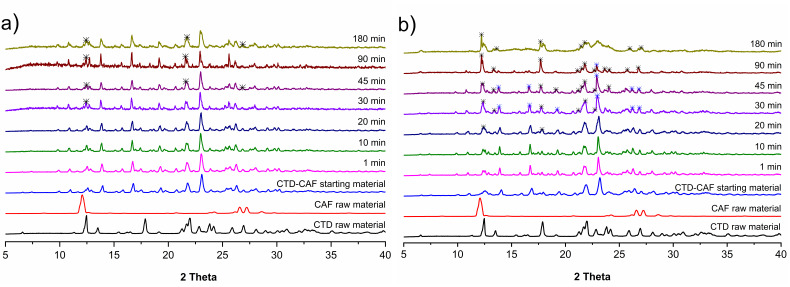
PXRD patterns of samples recovered from the CTD-CAF powder dissolution experiments of CTD-CAF under non-sink conditions in (**a**) HCl pH 1.2 and (**b**) PBS pH 6.8.

PXRD analyses of samples recovered after the non-sink dissolution experiments are shown in Figure 13. In the first diffraction patterns only peaks for the cocrystal are observed; however, at *t* = 30 min a characteristic peak for CTD-polymorph I appears at 2theta = 12.4°. Peaks for CTD increase then in number and intensity until the end of the experiments, where a mixture of characteristic peaks of CTD and CTD-CAF are observed for the dissolution studies at pH 1.2, while for pH 6.8 mostly CTD peaks are present.

**Figure 14:**
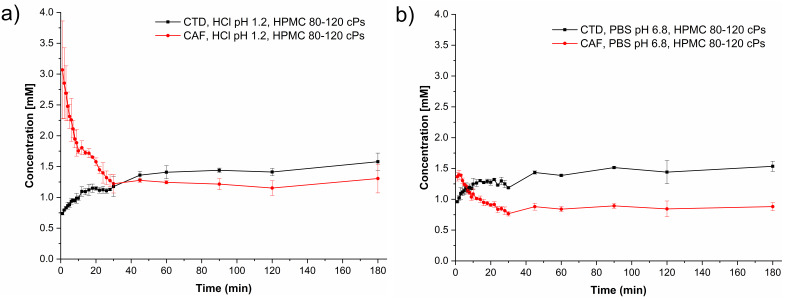
Dissolution profiles of CTD-CAF under non-sink conditions with HPMC 80–120 cPs predissolved at 0.5% (*w*/*v*) in (**a**) HCl pH 1.2 and (**b**) PBS pH 6.8. Dissolution profile lines are black for CTD and red for CAF. (mean ± SD, *n* = 3).

**Figure 15:**
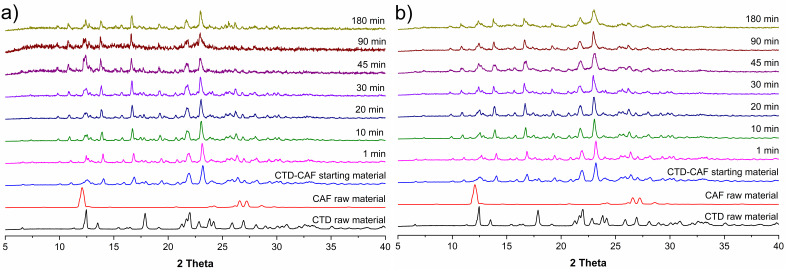
PXRD patterns of samples recovered from the powder dissolution experiments of CTD-CAF under non-sink conditions in the presence of HPMC 80–120 cPs pre-dissolved at 0.5% (*w*/*v*) in (**a**) HCl pH 1.2, and (**b**) PBS pH 6.8.

The cocrystal dissolution profiles under non-sink conditions at pH 1.2 and 6.8 in the presence of HPMC 80–120 cPs pre-dissolved at 0.5% (*w*/*v*) are presented in Figure 14. At acidic pH, the initial CTD concentration of 0.74 mM increased until reaching a plateau with a maximum concentration of 1.58 mM. A similar behavior is observed in the cocrystal dissolution profile at pH 6.8 with a maximum CTD concentration of 1.53 mM at the end of the experiment. By contrast, the CAF concentration from the dissolving cocrystal reached its maximum already at *t* = 1 min, decaying then in a lapse of 30 min to an approximately constant value of 1.22 mM at pH 1.2 and 0.77 mM at pH 6.8. PXRD analyses of the powder samples recovered from these dissolution experiments revealed that in this case the cocrystal phase remained unchanged. Peaks for CTD were not detected (Figure 15). On the contrary, dissolution experiments of a 2:1 physical mixture of CTD and CAF with pre-dissolved polymer (Figure S3c,d) gave profiles for CTD and CAF similar to the experiments without polymer and subsequent PXRD analysis revealed only peaks corresponding to CTD (Figure S4c,d).

### 3.9. Induced Precipitation Experiments

**Figure 16:**
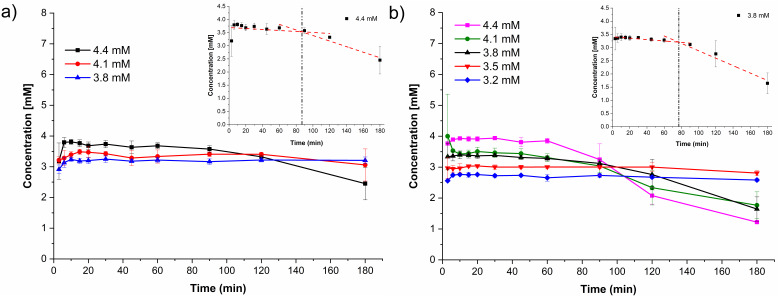
Induced precipitation profiles of CTD in (**a**) HCl pH 1.2 and (**b**) PBS pH 6.8 (mean ± SD, *n* = 3). The inserts in (**a**) and (**b**) shows the determination of the induction time (*t*_ind_).

Induced precipitation experiments were performed to estimate the CTD concentration critical for precipitation from the bulk phase (*C*_crit,BP_). This parameter is key to differentiate whether cocrystal solution-mediated phase transformation is occurring in the bulk phase (BP-SMPT) or at the particle surface (PS-SMPT), according to Omori et al. [71]. To initiate the experiments, an aliquot of the CTD stock solution was added to vials of stirred aqueous medium to obtain initial CTD concentrations of 3.8, 4.1, and 4.4 mM at pH 1.2 and 37 °C. At pH 6.8, five CTD solutions were tested with concentrations ranging from 3.2 mM to 4.4 mM. In each experiment, aliquots were taken at different time intervals (*t* = 1–180 min.) and filtered, whereupon the CTD concentration was quantified by HPLC. The resulting induced precipitation profiles for CTD at pH 1.2 and 6.8 are shown in Figure 16.

In some experiments, CTD crystallization/precipitation was observed by appearance of turbidity in the solution. Additionally, the induction time *t*_ind_ was estimated from the intercepts of the initial CTD concentration and the precipitation lines, as indicated in the inserts in Figure 16. At pH 1.2, *C*_crit,BP_ was 4.4 mM with an induction time of *t*_ind_ = 87 min. At pH 6.8, the critical value was 3.8 mM with *t*_ind_ = 77 min. Below these concentrations, CTD precipitation was not observed during the experiments. At pH 6.8 a linear correlation between the initial supersaturation ratio (*S*_ratio_ = *C*_CTD,t=0_/*S*_drug_) and the precipitation induction time (*t*_ind_) is observed (Table S3, Figure S5), which is indicative of a homogeneous nucleation process [72].

### 3.10. Dissolution Studies under Sink Conditions

**Figure 17:**
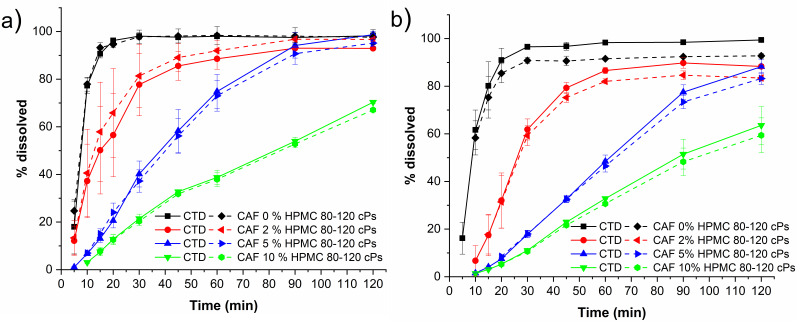
Dissolution profiles under sink conditions of capsules containing cocrystal equivalent to 50 mg of CTD and HPMC 80–120 cPs at 0, 2, 5, and 10% (*w*/*w*) in (**a**) HCl pH 1.2 and (**b**) PBS pH 6.8 (mean ± SD, *n* = 3). Note: solid lines correspond to CTD profiles, and dashed lines correspond to CAF profiles.

To evaluate the performance of a solid pre-formulation, the dissolution behavior of solid mixtures containing cocrystal CTD-CAF and the polymer explored herein at different *w*/*w* ratios were assessed under sink conditions using USP dissolution apparatus 1. In Figure 17 are presented the dissolution profiles of capsules containing 64.3 mg of cocrystal (equivalent to 50 mg of CTD) and different amounts of HPMC 80–120 cPs covering 0, 2, 5, and 10% (*w*/*w*) of a 141.5 mg tablet at pH 1.2 and pH 6.8 (see Table 1). Dissolution of the capsule with the bare cocrystal reached almost 100% of CTD and CAF dissolved within 30 min in both media. However, for the mixtures with polymer, dissolution of the cocrystal occurred at a slower rate, giving approx. 80, 40, and 20% of CTD and CAF in solution after 30 min for the mixtures using 2, 5, and 10% (*w*/*w*) of the polymer, respectively. PXRD analyses of the solids recovered from the experiments with polymer at 10% (*w*/*w*) showed only peaks characteristic of the cocrystal (Figure S6).

**Figure 18:**
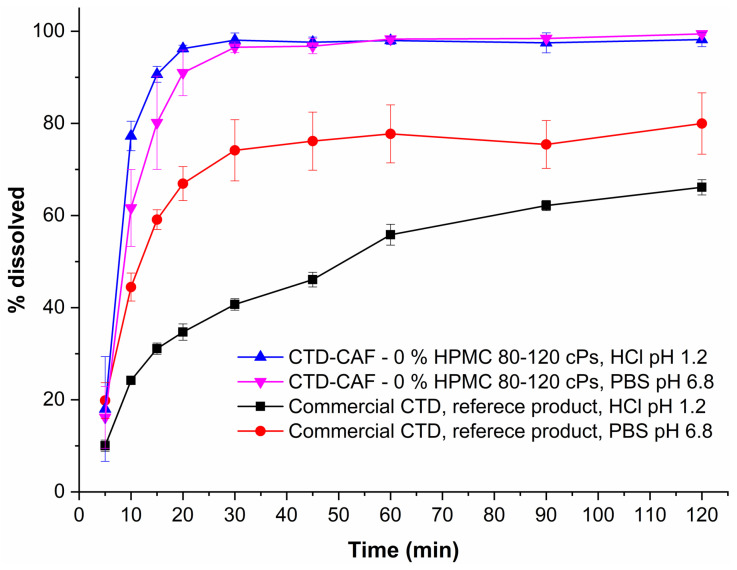
Dissolution profiles under sink conditions of capsules containing CTD-CAF powder (equivalent to 50 mg of CTD) and a commercial CTD reference product powder (mean ± SD, *n* = 3).

For comparative purposes, dissolution experiments under sink conditions were performed also using a commercial CTD reference product. Tablets of this product were ground in an agate mortar and passed through a sieve mesh 200 to obtain the powders required to fill the capsules. For the experiments, a weight equivalent to 50 mg of CTD was introduced in hard capsules size 4. Dissolution profiles of capsules containing cocrystal CTD-CAF without polymer and the commercial CTD reference product powder are shown in Figure 18. Interestingly, the capsules containing the cocrystal showed faster dissolution than the commercial CTD product in both media (pH 1.2 and 6.8). After 30 min, CTD was fully dissolved from the cocrystalline form while the commercial CTD product provided only 74% at pH 6.8 and 41% at pH 1.2. Moreover, for the commercial CTD powders, dissolution reached only approx. 80% at 120 min in PBS pH 6.8 and 66% in HCl pH 1.2, leaving solid residues. The PXRD patterns for the residual solids showed peaks corresponding to CTD (Figure S7).

## 4. Discussion

There are three known polymorphs of CTD and one chloroform solvate reported in the literature. Polymorph I is the most stable form and is the solid present in the commercially available formulations; however, this phase is class IV according to the BCS due to its low solubility and permeability, which leads to limited bioavailability and dose-dependent side effects. Therefore, it is desirable to generate alternative solid forms with better solubility and dissolution behavior, using a simple preparation method. The cocrystal of CTD with caffeine (CTD-CAF) presented herein was prepared by the slurry method using ethanol as solvent. Slurry procedures have been shown to generate multicomponent crystalline materials [43,73,74,75] even at large scale as reported for industrially relevant drugs such as carbamazepine [76] and telmisartan [77]. CTD-CAF is the first cocrystal of CTD fully characterized by diverse physical and spectroscopic techniques, including elucidation of the molecular and crystal structure by SCXRD analysis. The crystallographic analysis of the solid confirmed the 2:1 CTD-CAF composition and revealed the formation of a lattice inclusion compound, in which the CAF molecules are embedded in channels of a 3D network of CTD molecules. All hydrogen bonding motifs observed in CTD-polymorph I are present also in the crystal structure of CTD-CAF (motifs **I**–**IV**, Figure 4), and there are additional interactions between CAF and CTD entities through hydrogen bonds **V**–**VI**. Interestingly, the thermal stability of the cocrystal in relation to the melting point is lower (197 °C) compared to CTD-polymorph I (211 °C); however, considering the enthalpy of fusion the cocrystal consumes more energy (Δ_fus_H^o^ = 95.8 kJ mol^−1^ vs. 75.5 kJ mol^−1^ for CTD), probably due to stronger host-guest binding through the O-H⋯O and N-H⋯N bonds described above. This extra stability might explain why CAF release was not observed as well-defined thermal event in the TGA after fusion of the cocrystal solid.

### 4.1. Solubility of CTD-CAF Cocrystal and Performance under Non-Sink Conditions

Pharmaceutical cocrystals are an alternative to overcome physicochemical limitations of current APIs formulated as oral dosage forms. Particularly, solubility and dissolution behavior are of utmost importance given the relationship with drug absorption and bioavailability. Coformer selection is key to modulate the cocrystal solubility because a favorable solvation energy can drive also the solubilization of the API [44,78,79,80]. CAF (67.8 mM at 20 °C [81]) is 194-fold more soluble than CTD in water (0.35 mM at 20 °C [10]); therefore, we envisioned that a combination of CTD and CAF can produce a cocrystal with better solubility. However, prediction of the dissolution behavior is always somewhat uncertain given that a cocrystal solubility advantage also presents a risk for conversion to a less soluble form.

Our experiments showed that solid CTD-CAF suffers of phase transformation upon contact with aqueous solutions, as previously observed for various other pharmaceutical cocrystals composed of APIs having low solubility and water soluble coformers. This occurs as consequence of enhanced dissolution producing drug concentrations above saturation (i.e., supersaturation), and ultimately, driving precipitation of the less soluble solid form. Thus, the cocrystal solubility (*S*_CC_) was established from the coformer and CTD concentrations at the eutectic point using Equation (1), affording 2.00 mM at pH 1.2 and 2.05 mM at pH 6.8 (see Table 4). Considering the CTD solubility under the same conditions, solubility advantages of 2.91 (pH 1.2) and 3.19 (pH 6.8) were obtained. The solubility advantage (*SA*) represents the theoretical maximum supersaturation that the cocrystal could generate in a given medium, and hence, *SA* describes also the cocrystal potential for conversion to the less soluble drug [47]. The eutectic constant (*K*_eu_) is an additional thermodynamic indicator of cocrystal stability and using Equation (2) values of 12.3 (pH 1.2) and 16 (pH 6.8) were established for CTD-CAF. In cocrystals with 2:1 drug:coformer ratio the *K*_eu_ value is larger than 0.5, then the cocrystal is more soluble than the pristine drug [45]. Accordingly, the cocrystal CTD-CAF can generate drug supersaturation and the risk of precipitation from bulk solution is low given that *S*_CC_ was found below the critical value *C*_crit,BP_ (Table 4). Previously studied cocrystals with high *K*_eu_ and *SA* values generally displayed high supersaturation and rapid phase transformation [36,82,83].

The solubility advantage of cocrystal over drug is also noted by evaluating the drug and cocrystal dose/solubility ratios (*D*_0D_ and *D*_0CC_, according to Equations (4) and (5)). These values represent the solubility enhancement necessary to dissolve the prescribed dose of a medicament in the luminal volume of 250 mL. When *D*_0D_ > 1, the drug solubility will not be sufficient to dissolve the dose and a similar reasoning applies for other solid forms of the drug (cocrystals, solvates, amorphous forms, etc.). *D*_0D_ ≤ 1 indicates that the total dose will be dissolved in 250 mL of aqueous medium. According to our findings (see Table 4), *D*_0D_ is 0.859 and 0.92 (at pH 1.2 and 6.8), near to the borderline value of *D*_0D_ = 1, while the cocrystal yielded a *D*_0CC_ about 0.29, clearly indicating that the full dose will dissolve in the luminal volume [36].

Dissolution studies under non-sink conditions are used for assessing the performance of supersaturable solid formulations such as amorphous solid dispersions, salts, and cocrystals [34,84]. In this contribution the non-sink dissolution behavior of CTD and cocrystal CTD-CAF was studied at pH 1.2 and 6.8 (37 °C) in the absence and the presence of HPMC 80–120 cPs at 0.5% (*w*/*v*). For comparison, a physical mixture of CTD and CAF in 2:1 molar ratio was also tested. Under the conditions of these experiments, CTD polymorph I showed a constant solubility value of 0.65 mM, independently of the pH and the presence of polymer in the medium (Figure 11). Contrary to CTD in pure form, the cocrystal showed fast dissolution at pH 1.2 achieving a CTD supersaturation maximum of 2.36 at 1 min (*C*_max_ = 1.62 mM, Figure 12a). At pH 6.8, the supersaturation of CTD passed through a maximum of 2.23 at 20 min (*C*_max_ = 1.44 mM) (Figure 12b). However, at both pH the supersaturation state was not sustained and decayed over time due to precipitation of CTD-Polymorph I, as indicated by PXRD analysis of the powders recovered from the experiments (Figure 13). However, towards the end of these studies the CTD solubilization had not fully decayed to the same concentration as pure CTD because of the presence of residual cocrystal in the final powders (Figure 13).

The cocrystal dissolution advantage can be better appreciated by comparing the values of the Area Under the Curve (AUC, mM min), which are extracted from the dissolution graphs and gathered in Table 5. The AUC values are for three different time intervals: from 0 to 45 min (AUC_0–45_), from 45 to 180 min (AUC_45–180_) and for the entire profile (AUC_Total_). The main differences between the dissolution profiles of cocrystal CTD-CAF and pure CTD occurred during the first 45 min, where supersaturation was transiently sustained for CTD-CAF. The AUC_0–45_ of the cocrystal (e.g., AUC_0–45_, CC(1.2) = 55.1 mM min) has almost twice the value of pure CTD (AUC_0–45_, CTD(1.2) = 28.0 mM min) or the physical mixture of CTD and CAF (AUC_0–45_, PM(1.2) = 32.9 mM min). After 45 min, the effect originated from precipitation of CTD is more pronounced (Figure 13), and therefore, the differences in the AUC_45–180_ between CTD-CAF and CTD (or the physical mixture) are less pronounced (see Table 5). Comparing the entire profiles (AUC_Total_), the dissolution advantage of the cocrystal is 1.45 and 1.81-fold (at pH 1.2 and 6.8, respectively), compared to the pristine drug.

Ideally, the effect of drug supersaturation from a dissolving cocrystal should be maintained to assure maximum drug absorption. In our experiments, cocrystal CTD-CAF generated a transient supersaturation for 45 min, which is limited by the concomitant precipitation of the less soluble CTD polymorph I. A supersaturation state is a driving force for nucleation and crystal growth, and hence, to impede or delay this outcome, polymers acting as precipitation inhibitors have proven to be effective in sustaining drug solubilization in aqueous environments [85]. Hydrogen bond formation has been proposed as one of the mechanisms by promoting drug-polymer association to maintain high drug concentrations in supersaturable systems (i.e., amorphous forms or cocrystals). As seen from the crystal structures of CTD and CTD-CAF, the molecular structure of CTD has functional groups capable of donating and accepting hydrogen bonds (i.e., 3 donors and 5 acceptors). Because of this, cellulosic and non-cellulosic polymers of pharmaceutical grade (Table S2) containing different numbers of hydrogen bonding functionalities were selected to find a suitable precipitation inhibitor for CTD. Using the solvent-shift approach [35], the polymer that best inhibited CTD precipitation was HPMC 80–120 cPs (Figure 10). This hydroxypropyl methylcellulose contains a higher proportion of hydrogen bond acceptors than donors, and sustained CTD concentrations as high as 22.7 mM (compared to 0.687 mM for solubility of CTD polymorph I at pH 1.2). However, a direct correlation between the number and type of hydrogen bond functions and the polymer capacity for inhibiting CTD precipitation could not be deduced from the solvent-shift results (Table S2, Figure 10). Polymers such as Kollidon^®^ 25 or Kollidon^®^ 90 which carry only an acceptor group per repeating unit had better performance than those with a large number of donors and acceptors (e.g., HPMC 80,000 or HPMC 370,000), underscoring the importance of other effects influencing the drug-polymer association.

In continuation of the above-described powder dissolution studies under non-sink conditions, experiments were performed also with HPMC 80–120 cPs pre-dissolved at 0.5% (*w*/*v*) in pH 1.2 and 6.8. The drug concentration-time profiles are presented in Figure 14. In the presence of the polymer, the supersaturated drug concentrations obtained from CTD-CAF were maintained during the time course of the experiments, generating the so-called spring-parachute effect, in similar fashion to previously studied combinations of cocrystals and polymers [15,83]. PXRD analyses of the solids recovered during the dissolution studies showed that during these experiments only solid CTD-CAF was in contact with the dissolution media (Figure 15). Interestingly, in the same experiments the CAF concentrations raised immediately to a maximum followed by a continuous decrease over time. A similar effect was observed by Alhalaweh, Ali, and Velaga [83] for an indomethacin-saccharin cocrystal, where the presence of PVP pre-dissolved at 2% (*w*/*v*) at pH 3 improved the indomethacin dissolution and decreased the saccharin dissolution below its solubility value.

The AUC values for the dissolution experiments of CTD-CAF in the presence and absence of the polymer are statistically different (*p* < 0.05) (Table 5, Table S4). The AUC_0ߝ45_ values were slightly higher in the absence than in the presence of the polymer. The main differences occur after 45 min, when CTD precipitation is reduced with the polymer inhibiting nucleation and/or crystal growth. At pH 1.2, the AUC_45–180_ value for the cocrystal without polymer is 114.7 mM min versus 196 mM min with the polymer. Overall, in the presence of the polymer the dissolution advantage of the cocrystal reached a constant 2.07- and 2.06-fold increase over the pristine drug (at pH 1.2 and 6.8, respectively).

### 4.2. Study of the Solution-Mediated Phase Transformation (SMPT) Mechanism

Solution-mediated phase transformation (SMPT) consists in the generation of a thermodynamically more stable and less soluble solid during the dissolution of a metastable phase that can arise from supersaturated bulk solutions (BP-SMTP) or at the surface of a dissolving solid (PS-SMTP) [86]. For pharmaceutical cocrystals, BP-SMPT occurs once the cocrystal has dissolved to a degree that the drug molecules (considered as the low-solubility component) exceed the critical concentration (*C*_crit_), whereupon precipitation of the less soluble phase takes place. In PS-SMPT, cocrystal dissolution and overrun of the critical concentration (*C*_crit_) happen locally, at the cocrystal particle surface, and before the drug molecules diffuse into the bulk phase, so that the less soluble phase is deposited on the cocrystal particle surface [71].

In the powder dissolution experiments under non-sink conditions without polymer, cocrystal CTD-CAF gave maximum supersaturated concentrations of 1.62 mM and 1.44 mM at pH 1.2 and 6.8, respectively (Figure 12). These concentrations are between the CTD solubility (*S*_drug_ = 0.687 mM and 0.643 mM, at pH 1.2 and 6.8, respectively) and the critical concentration determined in the induced precipitation studies (*C*_crit_ = 4.4 mM at pH 1.2 and 3.8 mM at pH 6.8). Accordingly, the SMPT observed in our studies for CTD-CAF during the powder dissolution experiments must have occurred on the particle surface (PS-SMPT). Similar results were reported for carbamazepine cocrystals [47,71]. CTD polymorph I was reported as a slow nucleating and fast growing crystalline solid by Rathi et al. [87]. Our results from the CTD-induced precipitation experiments confirmed these findings (Figure 16); even so, for the case of CTD-CAF, the PS-SMPT may be facilitated by the similarity between the structural motifs observed in the cocrystal CTD-CAF and CTD polymorph I (Table 3). The presence of the polymer pre-dissolved in the dissolution medium clearly affects the PS-SMPT of the dissolving cocrystal, because CTD supersaturated concentrations are sustained during the time course of the non-sink experiments (Figure 14). Hence, it seems that nucleation and/or growth is inhibited by the polymer locally at the surface.

### 4.3. Performance of CTD-CAF Cocrystal Pre-Formulations

Drugs require formulation to optimize the stability, efficacy, and safety properties required for oral administration. In a recent report, Kavanagh, et al. [42] list some examples of formulations with cocrystals, of which some products are already commercially available. Since pre-formulation studies in the phase of drug-development are useful for optimizing drug performance in a later stage, dissolution experiments under sink conditions were conducted for CTD-CAF with and without polymer using the USP apparatus 1 to avoid capsule flotation.

Capsules containing only CTD-CAF (0% polymer) showed a very fast dissolution rate, where more than 85% of the dose was dissolved within 15 min and 100% after 30 min, in both dissolution media (pH 1.2 and 6.8, Figure 17). No solids were observed at the end of the tests. Cocrystal capsules with 2, 5, 10% *w*/*w* of HPMC 80–120 cPs induced a slower CTD release-dissolution with increasing polymer concentration in the formulation (Figure 17). Moreover, CTD-CAF capsules formulated with HPMC 80–120 cPs at 10% (*w*/*w*) contained residual solid at the end of the dissolution experiment, for which PXRD analysis revealed that the CTD-CAF cocrystal phase was conserved. In these experiments the concentration-time profile for CAF is similar to CTD.

On the other hand, the commercial CTD reference product powder showed a pH-dependent dissolution profile (Figure 18); where at pH 1.2 only around 41% of CTD was dissolved after 30 min, increasing approximately to 74% at pH 6.8. At the end of the dissolution test (*t* = 120 min), only 66% and 80% of the CTD dose was dissolved (at pH 1.2 and 6.8, respectively). PXRD analyses of the solid residues confirmed the presence of CTD polymorph I in addition to other amorphous components in the formulation (Figure S7).

These results are promising, showing that the CTD-CAF cocrystal allows good control over the dissolution process and is pH-independent, achieving 100% CTD dissolution within 30 min even in the absence of additives. This indicates that immediate release dosage forms could be developed. In addition, CTD controlled-release dosage forms might become available in the presence of suitable polymers.

## 5. Conclusions

The cocrystal of chlorthalidone with the Generally Recognized As Safe (GRAS) coformer caffeine in 2:1 ratio molar presented in this work was prepared by the standard slurry technique using the eco-friendly solvent ethanol and characterized by PXRD, TG-DSC, and IR spectroscopic analysis. From the DSC analysis, a melting point lower than for CTD polymorph I was found. The structural studies were accomplished by SCXRD analysis, showing two CTD and one CAF molecule in the asymmetric unit. The cocrystal CTD-CAF and CTD polymorph I share all four principal supramolecular hydrogen bonding motifs, which are less represented in CTD forms II and III and the chloroform solvate. In the cocrystal, the CTD entities are organized in a 3D network comprising channels, in which pairs of CAF molecules are embedded.

Cocrystal CTD-CAF showed physical solid phase stability under temperature and relative humidity stress conditions, which made it viable to continue with pharmaceutical development studies. Powder dissolution studies of the cocrystal under non-sink conditions revealed CTD supersaturation states and a threefold solubility advantage compared to the pristine drug independently of the pH of the aqueous medium. Subsequent studies showed that CTD-CAF cocrystals transform to CTD form I according to a solution mediated phase transformation at the particle surface. Notably, CTD precipitation inhibition could be achieved in the presence of pre-dissolved polymer HPMC 80–120 cPs.

Finally, dissolution experiments under sink conditions unveiled that the CTD-CAF cocrystal induced, in pH-independent manner, faster and more complete CTD dissolution when compared to commercial tablets of CTD. However, formulations containing cocrystal CTD-CAF and the polymer HPMC 80–120 cPs showed decreased CTD release-dissolution behavior, compared to the cocrystal without polymer, making apparent the need of further pre-formulation studies to establish the right conditions to maintain the CTD supersaturation induced from the cocrystal dissolution.

Since the cocrystal has increased solubility and dissolution performance compared to a commercial CTD product, the possibility of conducting bioavailability studies using a lower CTD dose to obtain the same therapeutic response arises which should diminish adverse effects.

## Figures and Tables

**Table 1:** Compositions (in mg) of CTD-CAF cocrystal capsule formulations **1**–**4** with and without HPMC 80–120 cPs.

Formulation	1	2	3	4
CTD-CAF ^a^	64.3	64.3	64.3	64.3
HPMC 80–120 cPs ^b^	-	2.8	7.1	14.2

^a^ According to the molecular weights of CTD (338.77 g/mol), CAF (194.19 g/mol), and CTD-CAF in 2:1 stoichiometry ratio (871.73 g/mol), 64.33 mg of CTD-CAF are equivalent to 50 mg of CTD. ^b^ The amount of polymer corresponds to 0, 2, 5 and 10% *w*/*w*, considering as 100% the average weight of the commercial tablets (141.5 mg).

**Table 2:** Crystallographic data of the chlorthalidone-caffeine cocrystal (CTD-CAF).

Parameter	Value
Formula	C_36_H_32_Cl_2_N_8_O_10_S_2_
*MW* (g mol^−1^)	871.71
*T* (K)	298
Crystal system	triclinic
Space group	*P*-1
*a* (Å)	8.3218(5)
*b* (Å)	14.6139(9)
*c* (Å)	16.2923(10)
*α* (deg)	72.529(3)
*β* (deg)	81.128(3)
*γ* (deg)	86.070(3)
*Volume* (Å^3^)	1866.8(2)
*Z*	2
*ρ*_calc_ (g cm^−3^)	1.551
*μ* (mm^−1^)	3.225
*R*_1_ (*I* ≥ 2σ(*I*))	0.0550
*wR*_2_ (all data)	0.1568
*GOF*	1.029

**Table 3:** Hydrogen bonding motifs common to the crystal structures of cocrystal CTD-CAF and the crystalline solid forms reported of CTD.

Compound	Motif				
	I	II	III	IV	Reference
CTD-CAF	✓	✓	✓	✓	This work
CTD polymorph I	✓	✓	✓	✓	[23]
CTD polymorph II	✓	-	-	-	[24]
CTD polymorph III	✓	-	✓	-	[23]
CTD chloroform solvate	-	✓	-	-	[25]

**Table 4:** Cocrystal and Drug Solubility Data ^a^.

Dissolution Medium	*S*_drug_ [mM]	*S*_CC_ [mM]	*K*_eu_	*SA*	*D*_0D_	*D*_0CC_
HCl pH 1.2	0.687 ± 0.005	2.00 ± 0.03	12.3 ± 0.6	2.91 ± 0.05	0.859 ± 0.006	0.295 ± 0.004
PBS pH 6.8	0.643 ± 0.007	2.05 ± 0.05	16 ± 1	3.19 ± 0.09	0.92 ± 0.01	0.288 ± 0.007

^a^*S*_drug_ = solubility of CTD (polymorph I); *S*_CC_ = cocrystal solubility; *K*_eu_ = cocrystal eutectic constant; *SA* = solubility advantage; *D*_0D_ = drug dose/solubility ratio, and *D*_0CC_ = cocrystal dose/solubility ratio. *n* = 3 ± SD. *T* = 37 °C. A value of 0.59 mM for *C*_dose_ was used in the estimation of *D*_0D_ and *D*_0CC_.

**Table 5:** Area Under the Curve (AUC, mM min) for the dissolution under non-sink conditions of CTD starting from CTD polymorph I, a physical mixture of CTD and CAF and cocrystal CTD-CAF.

Solid Form (Dissolution Media) ^a^	AUC_0–45_^b^	AUC_45–180_^b^	AUC_Total_^b^	(AUC_Total, PM or CC_/AUC_Total, CTD_)
CTD (1.2)	28.0	88.6	116.6	-
CTD (6.8)	28.2	86.2	114.4	-
CTD (1.2, HPMC)	27.5	91.0	118.5	-
CTD (6.8, HPMC)	28.9	94.0	122.9	-
PM (1.2)	32.9	98.8	131.7	1.12
PM (6.8)	32.3	99.3	131.6	1.15
PM (1.2, HPMC)	34.4	98.5	132.9	1.12
PM (6.8, HPMC)	31.5	108.6	140.1	1.13
CC (1.2)	55.1	114.7	169.8	1.45
CC (6.8)	57.2	151.0	208.2	1.81
CC (1.2, HPMC)	49.4	196.0	245.4	2.07
CC (6.8, HPMC)	55.3	198.2	253.5	2.06

^a^ In parenthesis are indicated pH of the dissolution medium, and in case, the presence of HPMC 80–120 cPs at 0.5% *w*/*v* in. CC = cocrystal CTD-CAF (2:1). PM = physical mixture of CTD and CAF in 2:1 ratio. ^b^ AUC is given at three different time intervals: from 0 to 45 min (AUC_0–45_), from 45 to 180 min (AUC_45–180_) and for the entire profile (AUC_Total_).

## Supplementary Materials

The following supporting information can be downloaded at: https://www.mdpi.com/article/10.3390/pharmaceutics14020334/s1, Figure S1. PXRD patterns for the respective polymorphs reported in the CSD (Version 2020.3.0) of (a) CTD and (b) CAF. Note: Asterisks indicate peaks identical to the corresponding raw material. Table S1. Intermolecular hydrogen bonding interactions for cocrystal CTD-CAF. Table S2. Polymers used in this study and some relevant properties. Figure S2. PXRD patterns of solids recovered from powder dissolution experiments under non-sink conditions of CTD in (a) HCl pH 1.2, (b) PBS pH 6.8, (c) HCl pH 1.2 with HPMC 80–120 cPs pre-dissolved at 0.5% (*w*/*v*), and (d) PBS pH 6.8 with HPMC 80–120 cPs pre-dissolved at 0.5% (*w*/*v*). Figure S3. Dissolution profiles under non-sink conditions of physical mixtures containing CTD and CAF in 2:1 molar ratio in (a) HCl pH 1.2, (b) PBS pH 6.8, (c) HCl pH 1.2 with HPMC 80–120 cPs pre-dissolved at 0.5% (*w*/*v*), and (d) PBS pH 6.8 with HPMC 80–120 cPs pre-dissolved at 0.5% (*w*/*v*). Figure S4. PXRD patterns from solids recovered from powder dissolution experiments under non-sink conditions of physical mixtures of CTD and CAF in 2:1 molar ratio in (a) HCl pH 1.2, (b) PBS pH 6.8, (c) HCl pH 1.2 with HPMC 80–120 cPs pre-dissolved at 0.5% (*w*/*v*), and (d) PBS pH 6.8 with HPMC 80–120 cPs pre-dissolved at 0.5% (*w*/*v*). Table S3. Induction time (*t*_ind_), supersaturation ratio (*S*_ratio_), and data required to determine *t*_ind_ and *S*_ratio_ correlation for induced precipitation experiments in PBS pH 6.8. Figure S5. Correlation between the induction time (*t*_ind_) and the level of CTD supersaturation (*S*_ratio_). Figure S6. PXRD patterns of residual solids from the dissolution experiments of formulations containing CTD-CAF and HPMC 80–120 cPs. Figure S7. PXRD patterns of residual solids from dissolution experiments of commercial CTD reference product powder in capsules in HCl pH 1.2 and PBS pH 6.8. Table S4. Relevant results of the Two-Way ANOVA and Tukey Tests, at 0.05 significance.
