# Supplementary material for: Tailoring Chlorthalidone Aqueous Solubility by Cocrystallization: Stability and Dissolution Behavior of a Novel Chlorthalidone-Caffeine Cocrystal
Source: Pharmaceutics. 2022 Jan 30;14(2):334. doi: 10.3390/pharmaceutics14020334 (PMC8876770; doi:10.3390/pharmaceutics14020334)
Supplement: Supplementary file 1 [file pharmaceutics-14-00334-s001.zip › pharmaceutics-1558824-supplementary.pdf]

pharmaceutics

MDPI

Supplementary Materials: Tailoring Chlorthalidone Aqueous Solubility by Cocrystallization: Stability and Dissolution Behavior of a Novel Chlorthalidone-Caffeine Cocrystal

Christian Rodríguez-Ruiz, Pedro Montes-Tolentino, Jorge Guillermo Domínguez-Chávez, Hugo Morales-Rojas, Herbert Höpfl and Dea Herrera-Ruiz

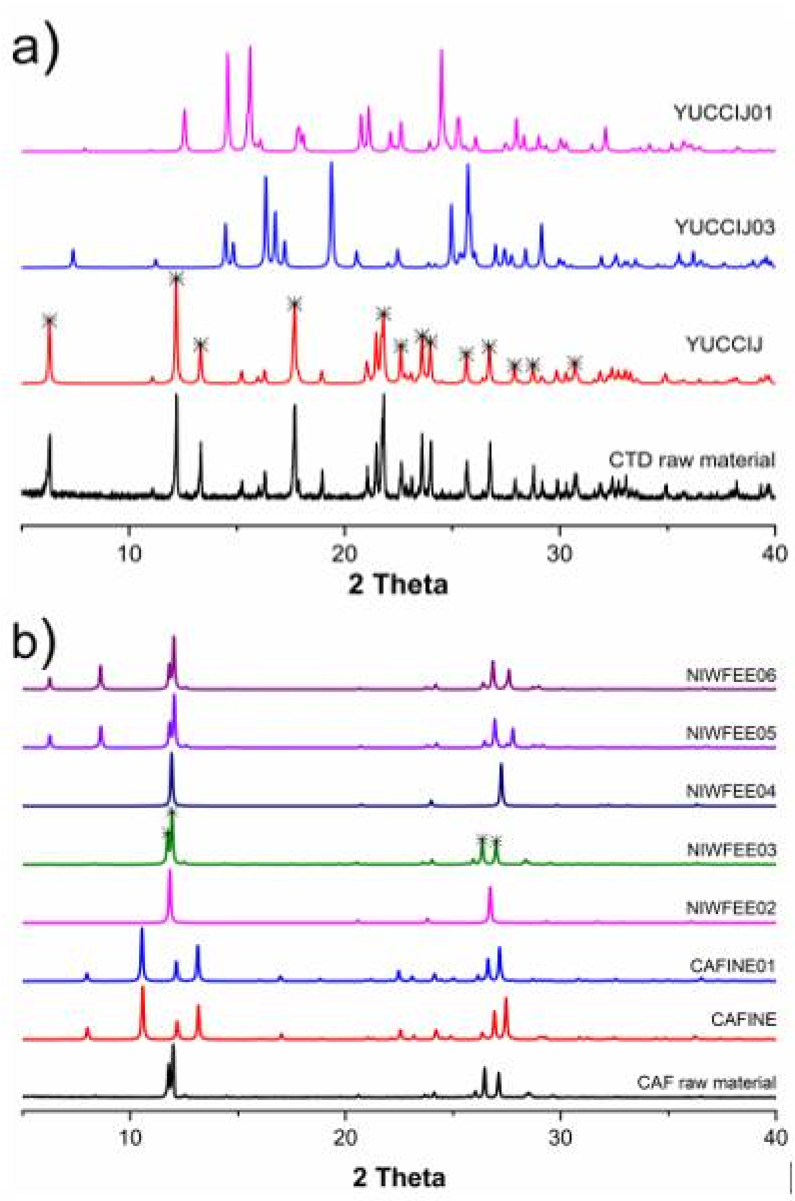

Figure S1. PXRD patterns for the respective polymorphs reported in the CSD (Version 2020.3.0) of (a) CTD and (b) CAF. Note: Asterisks indicate peaks identical to the corresponding raw material.

Table S1. Intermolecular hydrogen bonding interactions for cocrystal CTD-CAF.

Motif	H-Bond	D–H (Å)	H···A (Å)	D···A (Å)	∠DHA (deg)	Symmetr Operator
**I**	N1A-H1AA...O2B	0.91	2.30	3.188(4)	166	1+x, -1+y, z
	N1B-H1BB…O2A	0.91	2.43	3.313(4)	163	-1+x, 1+y, z
**II**	N2A-H2A…O4B	0.86	2.12	2.959(4)	165	x, y, z
	N2B-H2B…O4A	0.86	1.97	2.819(4)	171	x, y, z
**III**	O3B-H3B…O4B	0.82	1.96	2.778(3)	174	1-x, 1-y, 2-z
**IV**	N2A-H2A…O4B	0.86	2.12	2.959(4)	165	x, y, z
	O3B-H3B…O4B	0.82	1.96	2.778(3)	174	1-x, 1-y, 2-z
	N1A-H1AB…O3B	0.91	2.05	2.948(4)	168	1-x, 1-y, 2-z
**V**	O3A-H3A…O6	0.82	1.90	2.715(3)	172	x, y, z
**VI**	N1B-H1BA…N5	0.91	2.32	3.068(4)	139	1-x, 2-y, 1-z

Table S2. Polymers used in this study and some relevant properties.

Polymer	Molecular Structure	Average Molecular Weight	Viscosity	Hydrogen Bond (Donor-Acceptor)
Copovidone (Kollidon VA® 64 Fine)	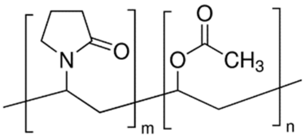	45,000–70,000	At concentrations less than 10% w/v, the viscosity is less than 10 mPas at 25 °C	D = 0 A= 2
Polyvinylpyrrolidone (Kollidon® 25)	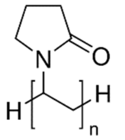	~34,000	3.5–5.5 mPas 10% w/v in H_2O at 20 °C	D = 0 A= 1
Polyvinylpyrrolidone (Kollidon® 90)		~1,300,000	300–700 mPas, 10% w/v in H_2O at 20 °C	
Poloxamer (Kolliphor® P 188)	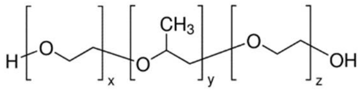	7,680–9,510	Not available	D = 1 A= 3
Poloxamer (Kolliphor® P 407)		9,840–14,600	Not available	
Hydroxypropyl cellulose (HPC 80,000)	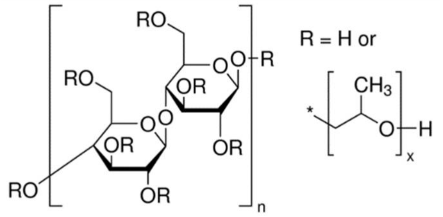	~80,000	250–800 cPs, 10 % (w/v) in H_2O at 25 C	D = 3 A= 6
Hydroxypropyl cellulose (HPC 370,000)		~370,000	150–400 cPs, 2 % (w/v) in H_2O at 25 °C	
Methylcellulose (Methocel® A15)	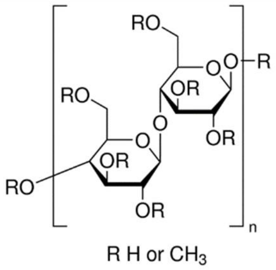	27.5-31.5% methoxy basis	12–18 mPa.s, 2% (w/v) in H_2O at 20 °C	D = 0 A= 5
Methylcellulose (Methocel® 60 H.G.)		28–30% methoxy basis	35–55 mPas, 2% (w/v) in H_2O at 20 °C	
Hydroxypropyl methylcellulose (Methocel® E5 LV)	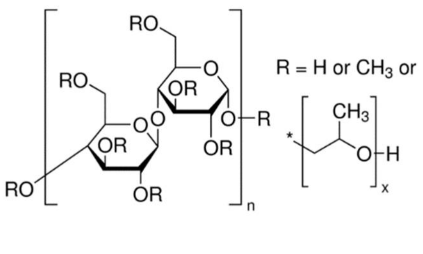	~10,000 Methoxy content 28 – 30 % hydroxypropoxy content 7–12%	4–6 mPas, 2% (w/v) in H_2O at 20 °C	D = 2 A= 6
Hydroxypropyl methylcellulose (Methocel® E15 LV)		~13,000 Methoxy content 28 – 30 % hydroxypropoxy content 7–12%	12–18 mPas, 2% (w/v) in H_2O at 20 °C	
Hydroxypropyl methylcellulose (Methocel® E50LV)		~20,000 Methoxy content 28 – 30 % hydroxypropoxy content 7–12%	40–60 mPas, 2% (w/v) in H_2O at 20 °C	
Hydroxypropyl methylcellulose (HPMC 80 – 120 cPs)		~26,000 Methoxy content 19 – 24% hydroxypropoxy content 7–12%	80–120 cP, 2% (w/v) in H_2O at 20 °C	

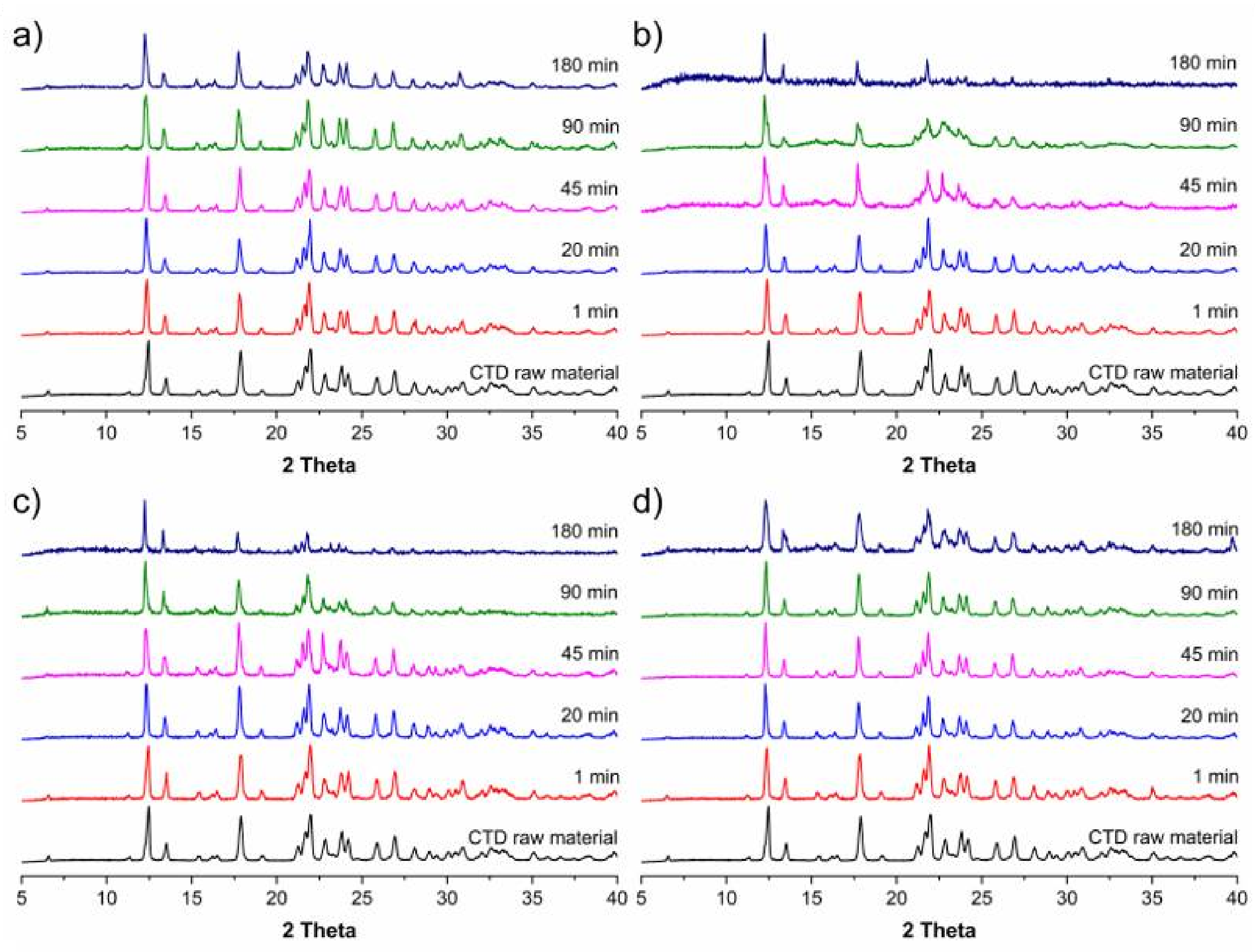

Figure S2. PXRD patterns of solids recovered from powder dissolution experiments under **non-sink** conditions of CTD in (a) HCl pH 1.2, (b) PBS pH 6.8, (c) HCl pH 1.2 with HPMC 80–120 cPs pre-dissolved at 0.5 % (**w/v**), and (d) PBS pH 6.8 with HPMC 80–120 cPs pre-dissolved at 0.5 % (**w/v**).

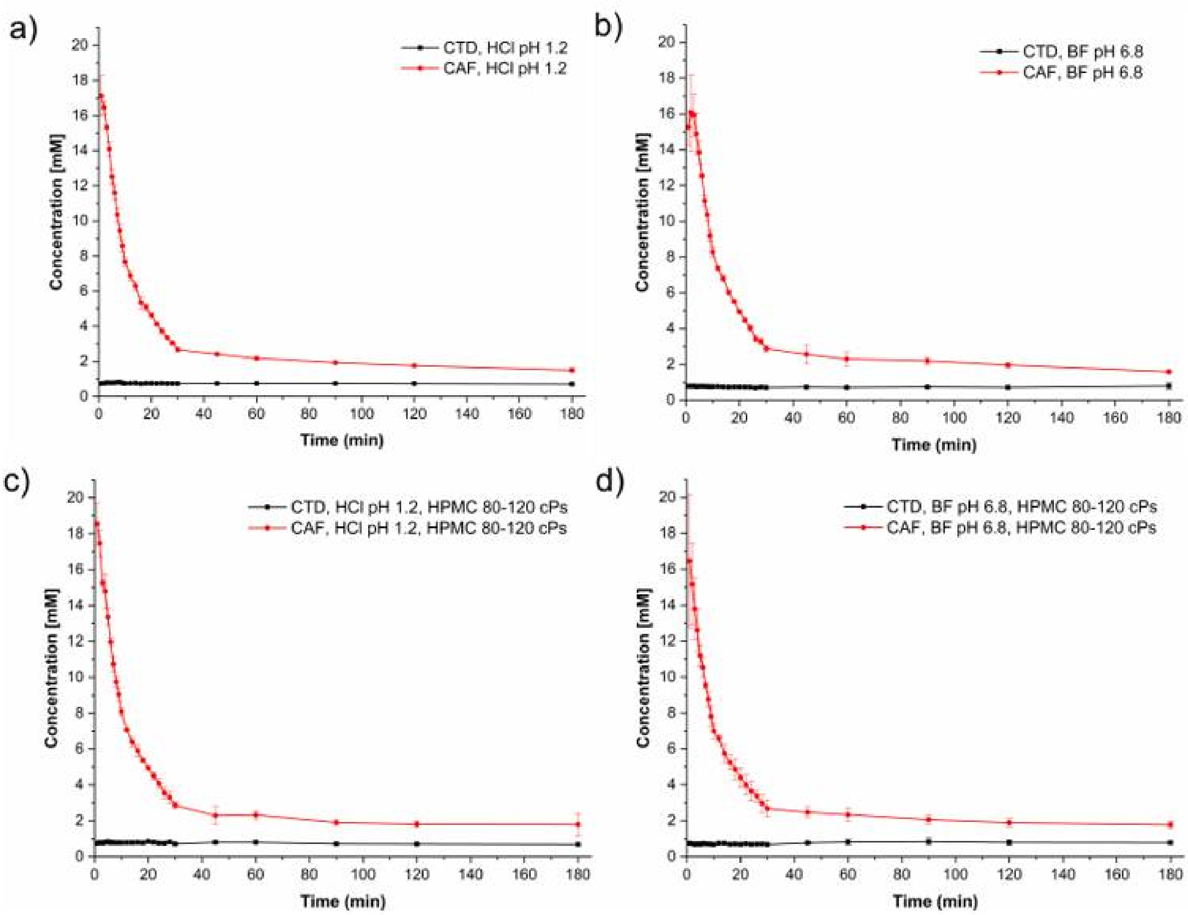

Figure S3. Dissolution profiles under **non-sink** conditions of physical mixtures containing CTD and CAF in 2:1 molar ratio in (a) HCl pH 1.2, (b) PBS pH 6.8, (c) HCl pH 1.2 with HPMC 80-120 cPs pre-dissolved at 0.5 % (**w/v**), and (d) PBS pH 6.8 with HPMC 80–120 cPs pre-dissolved at 0.5 % (**w/v**). Mean ± S.D., n = 3. Note: CAF profiles did not supersaturate. The amount of caffeine in 200 mg of physical mixture was 45 mg. Because of the high solubility of CAF, the coformer dissolved completely within the first minute of the experiments; therefore, the amount of CAF found in the medium decreased according to the dilution effect introduced by medium replacement. Hence, CAF concentrations decreased as observed in the dissolution profiles.

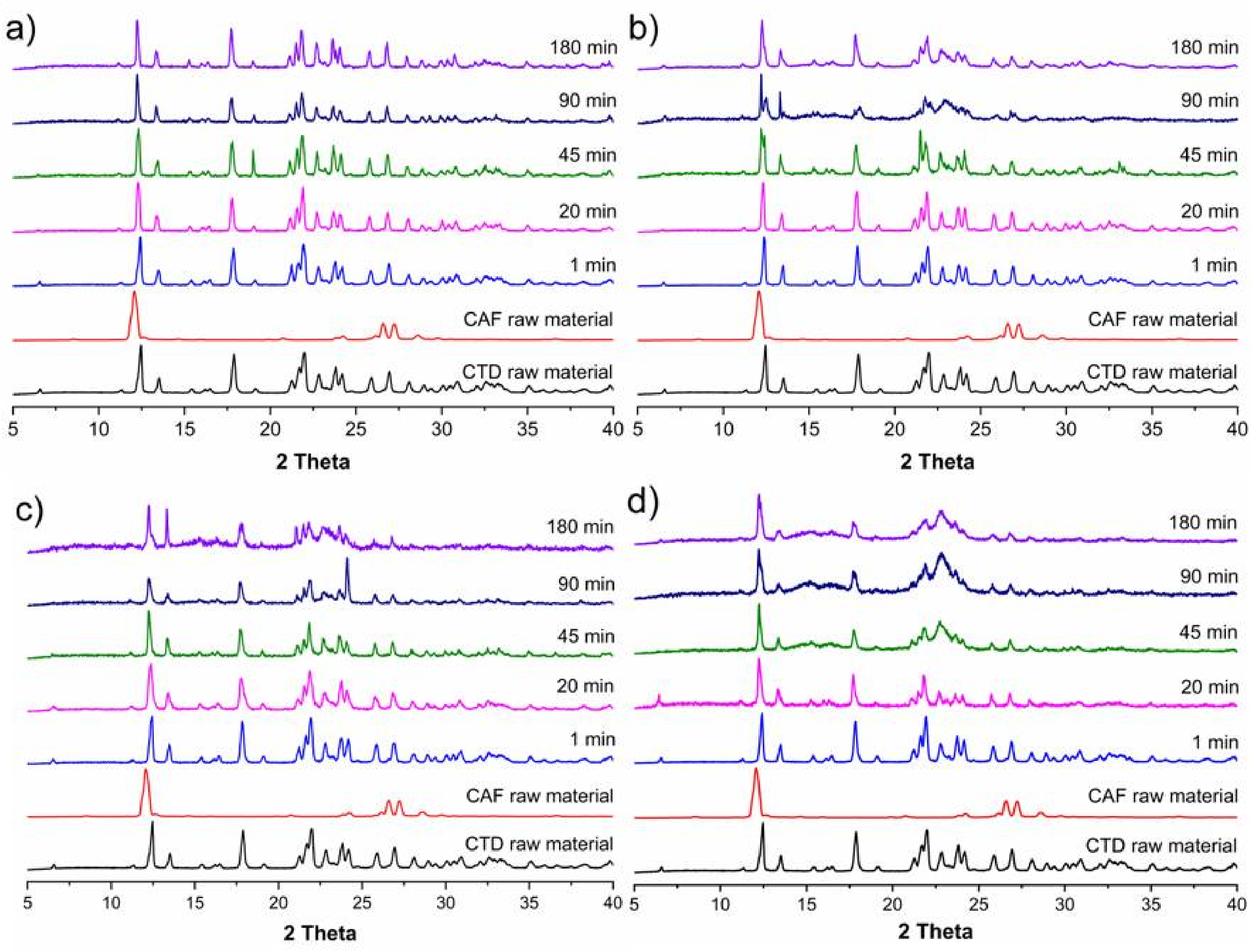

Figure S4. PXRD patterns from solids recovered from powder dissolution experiments under **non-sink** conditions of physical mixtures of CTD and CAF in 2:1 molar ratio in (a) HCl pH 1.2, (b) PBS pH 6.8, (c) HCl pH 1.2 with HPMC 80–120 cPs pre-dissolved at 0.5 % (**w/v**), and (d) PBS pH 6.8 with HPMC 80–120 cPs pre-dissolved at 0.5 % (**w/v**).

Table S3. Induction time (t_{ind}), supersaturation ratio (S_{ratio}), and data required to determine t_{ind} and S_{ratio} correlation for induced precipitation experiments in PBS pH 6.8.

$C_{CTD,\ t=0}$ (mM)	t_{ind} (min)	log t_{ind} (min)	S_{ratio} ($C_{CTD,\ t=0}/S_{drug}$)	log S_{ratio}	(log S_{ratio})$^{-2}$
3.8	77	1.90	5.9	0.77	1.68
4.1	63	1.80	6.4	0.80	1.55
4.4	50	1.70	6.9	0.83	1.44

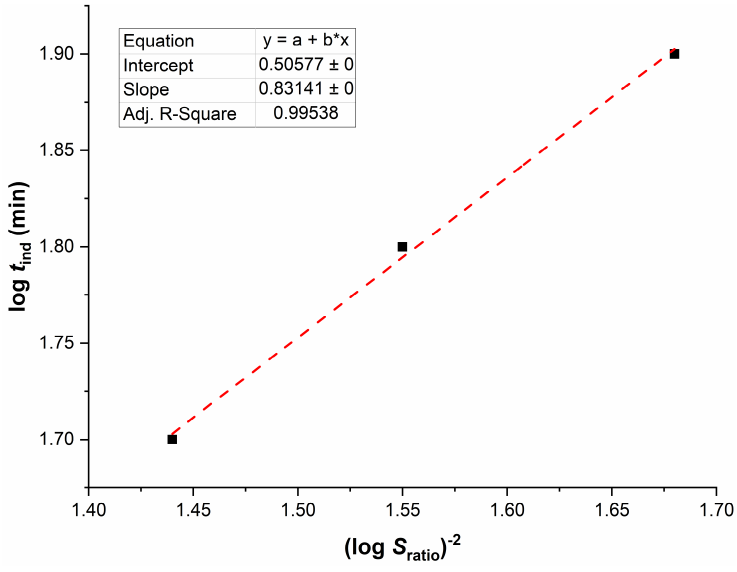

Figure S5. Correlation between the induction time (t_{ind}) and the level of CTD supersaturation (S_{ratio}).

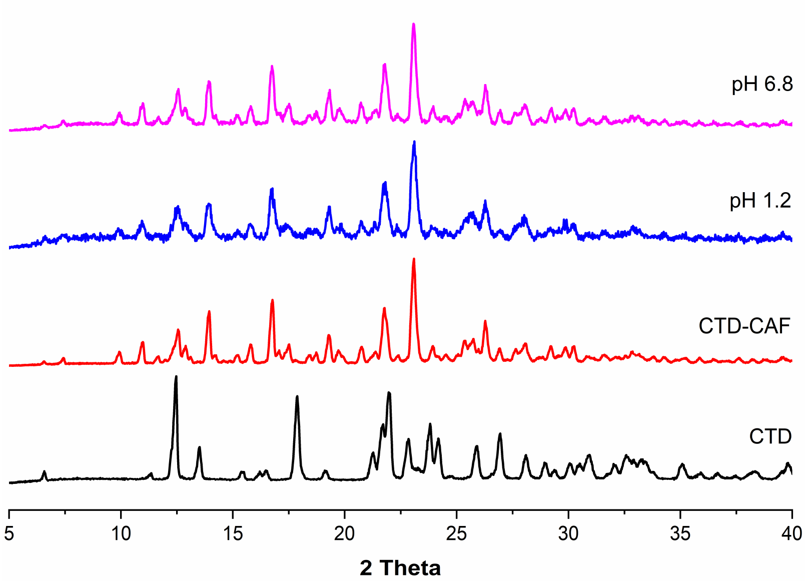

Figure S6. PXRD patterns of residual solids from the dissolution experiments of formulations containing CTD-CAF and HPMC 80-120 cPs. For comparison, PXRD patterns of CTD and CTD-CAF cocrystal before the experiments are also included.

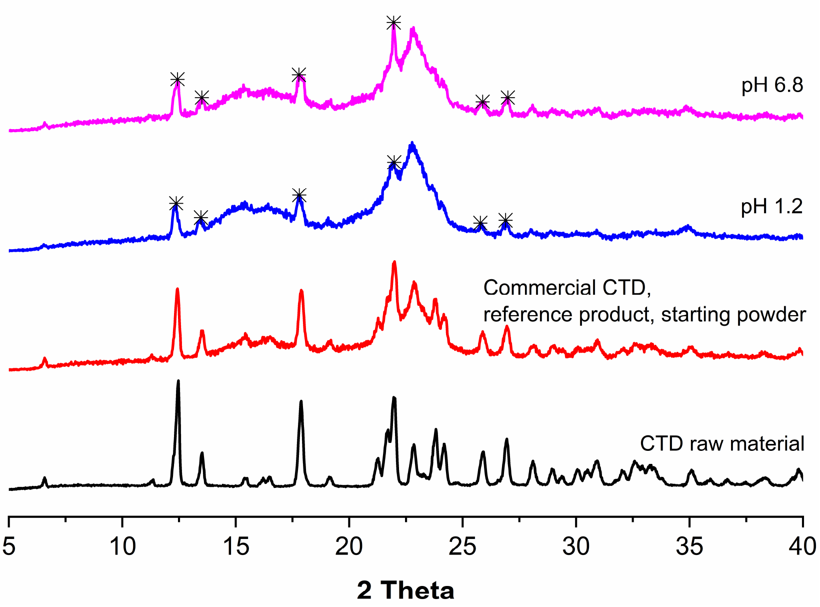

Figure S7. PXRD patterns of residual solids from dissolution experiments of commercial CTD reference product powder in capsules in HCl pH 1.2 and PBS pH 6.8. For comparison, PXRD patterns of CTD and commercial CTD product powder before the experiments also are shown. Note: asterisks indicate representative signals for CTD.

Table S4. Relevant results of the Two-Way ANOVA and Tukey Tests, at 0.05 significance.

A	B	A	B	Means Difference	Probability	Sig*
CTD	pH 6.8	CTD	pH 1.2	−2.23667	1	0
CTD	pH 1.2, HPMC	CTD	pH 1.2	1.88333	1	0
CTD	pH 6.8, HPMC	CTD	pH 6.8	8.49333	0.98556	0
CTD	pH 6.8, HPMC	CTD	pH 1.2, HPMC	4.37333	0.99996	0
MF CTD-CAF	pH 1.2	CTD	pH 1.2	15.10667	0.62587	0
MF CTD-CAF	pH 6.8	CTD	pH 6.8	17.15333	0.44939	0
MF CTD-CAF	pH 1.2, HPMC	CTD	pH 1.2	16.23333	0.52735	0
MF CTD-CAF	pH 6.8, HPMC	CTD	pH 6.8	25.69667	0.05325	0
MF CTD-CAF	pH 1.2, HPMC	CTD	pH 1.2, HPMC	14.35	0.69106	0
MF CTD-CAF	pH 6.8, HPMC	CTD	pH 6.8, HPMC	17.20333	0.44528	0
CTD-CAF	pH 1.2	CTD	pH 1.2	53.14667	6×10^{-6}	1
CTD-CAF	pH 6.8	CTD	pH 6.8	93.81	2×10^{-7}	1
CTD-CAF	pH 1.2, HPMC	CTD	pH 1.2	128.68667	6×10^{-8}	1
CTD-CAF	pH 6.8, HPMC	CTD	pH 6.8	139.05667	6×10^{-8}	1
CTD-CAF	pH 6.8	CTD-CAF	pH 1.2	38.42667	8.5×10^{-4}	1
CTD-CAF	pH 1.2, HPMC	CTD-CAF	pH 1.2	75.54	3×10^{-8}	1
CTD-CAF	pH 6.8, HPMC	CTD-CAF	pH 6.8	45.24667	8×10^{-5}	1
CTD-CAF	pH 6.8, HPMC	CTD-CAF	pH 1.2, HPMC	8.13333	0.98964	0

*Sig equal 1 indicates that the difference of the means is significant at the 0.05 level.

*Sig equal 0 indicates that the difference of the means is not significant at the 0.05 level.
